# Synthetic Process Development of (*R*)-(+)-1,2-Epoxy-5-hexene: An Important Chiral Building Block

**DOI:** 10.1021/acs.oprd.4c00101

**Published:** 2024-08-01

**Authors:** Daryl Guthrie, John M. Saathoff, Rajkumar Lalji Sahani, Aline Nunes De Souza, Daniel W. Cook, Samuel R. Hochstetler, Justina M. Burns, Roudabeh Sadat Moazeni-Pourasil, Janie Wierzbicki, Saeed Ahmad, G. Michael Laidlaw, B. Frank Gupton, Charles S. Shanahan, Douglas A. Klumpp, Limei Jin

**Affiliations:** Medicines for All Institute, Virginia Commonwealth University, Richmond, Virginia 23284-3068, United States

**Keywords:** lenacapavir, epoxide, chiral resolution, epichlorohydrin, DOE, process development, scalable

## Abstract

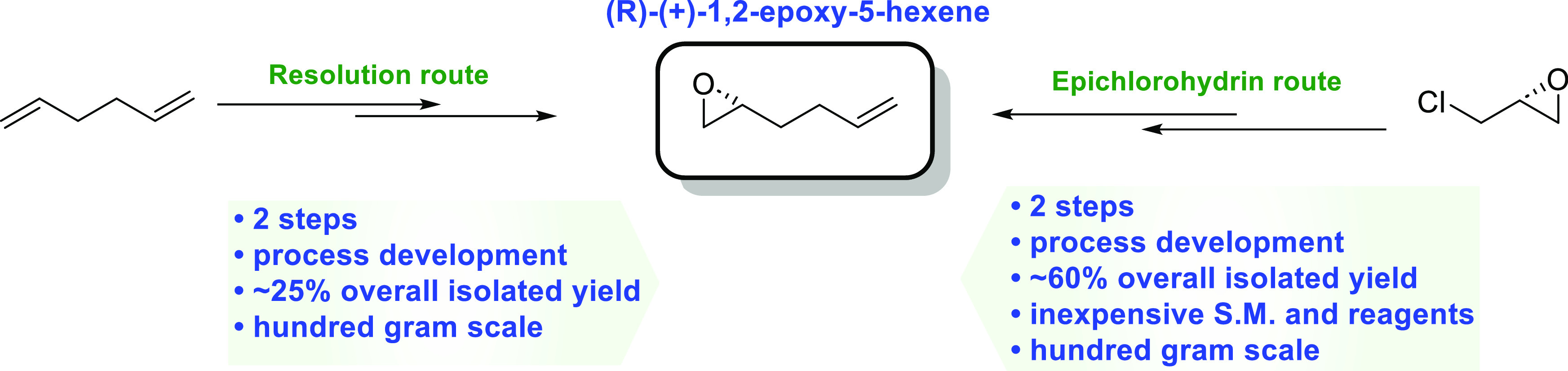

Herein, we describe two practical approaches to synthesize (*R*)-(+)-1,2-epoxy-5-hexene from inexpensive and readily available raw materials and reagents. The first approach is a two-step sequence, involving an epoxidation with *meta*-chloroperoxybenzoic acid (mCPBA) and a chiral resolution with (salen)Co(II), producing (*R*)-(+)-1,2-epoxy-5-hexene in 24–30% overall yield. The second approach utilizes readily available (*R*)-epichlorohydrin as the starting material and features an epoxide ring-opening reaction with allylMgCl and the NaOH-mediated ring closure reaction. Development of this two-step process affords *R*-(+)-1,2-epoxy-5-hexene in overall isolated yields of 55–60% with an exceptional purity profile. Both approaches have been successfully demonstrated on 100–200 g scales.

## 1 Introduction

**Scheme 1:**
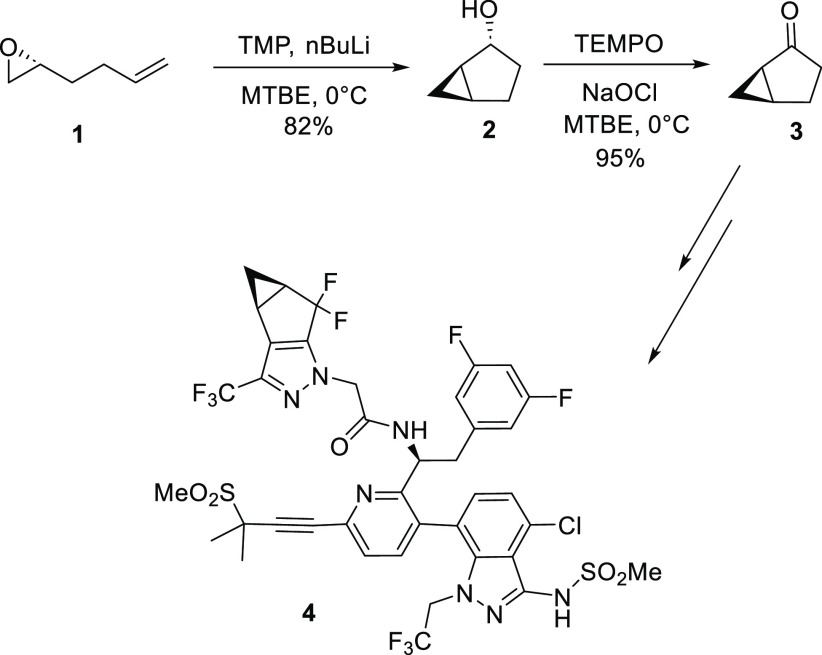
Reported Synthetic Route for the Synthesis of Compound **3** from Epoxide **1**

The epoxide (*R*)-1,2-epoxyhex-5-ene (**1**) is an important chiral building block. It has been used in a number of asymmetric synthetic methodologies, including the syntheses of chiral isothiazolidine-1,1-dioxides,^1^ benzoxathiazepine-1,1-dioxides,^2^ γ-butanolides,^3^ β-hydroxy morpholine amides,^4^ and varied alcohols. Several natural product syntheses have also utilized compound **1**, such as in the syntheses of (+)-gigantecin and (+)-14-deoxy-9-oxygigantecin,^5^ pyragonicin,^6^ amphidinolides C and F,^7,8^ and Sch725674 macrolactones.^9^ Biologically active compounds and pharmaceutical substances have also been prepared as single enantiomers from this chiral epoxide. For example, compound **1** is used in the synthesis of the C20–C26 fragment of the anticancer drug - Halaven.^10^ A kg-scale synthesis of the bicyclic ketone, (1*R*,5*S*)-bicyclo[3.1.0]hexan-2-one (**3**), has been reported, and this synthetic method begins with the chiral epoxide **1** (Scheme 1).^11^ In this transformation, the chiral epoxide is reacted with catalytic lithium 2,2,6,6-tetramethylpiperidide (TMP), which allows for insertion into the olefin group to provide the bicyclic alcohol (**2**). Oxidation of the alcohol gives compound **3** as a single enantiomer. This bicyclic ketone has been used to synthesize a potent series of cannabinoid receptor modulators and active pharmaceutical ingredients.^12,13^ We have successfully used compound **1** in our current synthesis of a fragment of lenacapavir **4**, an anti-HIV drug, as shown in Scheme 1.^14^

Previously, compound **1** was prepared by enantioselective hydrolytic ring opening of the racemic mixture. In chemistry developed by the Jacobsen group, the enantioenriched epoxide is obtained by hydrolytic kinetic resolution (HKR) involving a chiral (salen)Co (III) complex (the resolution route, Route 1).^15,16^ This method has been used by several other groups to prepare compound **1**.^7−10,17−21^ The chiral epoxide **1** has been prepared from the chiral epichlorohydrin (the epichlorohydrin route, Route 2).^22,23^ The reported routes offer initial pathways for the synthesis of (*R*)-1,2-epoxyhex-5-ene (**1**); however, the methods were only done on a small scale and require chromatographic purification. The racemic epoxide has been prepared by epoxidation of 1,5-hexadiene with *meta*-chloroperoxybenzoic acid (mCPBA)^24^ as well as other reagents.^25^ The racemic epoxide has also been synthesized from the chlorohydrin (itself obtained by the reaction of allyltrimethylsilane and epichlorohydrin with TiCl_4_).^26^

With the synthetic utility of compound **1**, we sought to develop a process that provides a scalable and economical route to this chiral epoxide. In this study, we focus on the process development of both the resolution route and the epichlorohydrin route. The results of our study are detailed in the following sections.

## 2 Results and Discussion

### 2.1 Synthesis of (*R*)-(+)-1,2-Epoxy-5-hexene through the Resolution Route (Route 1)

#### 2.1.1 Synthesis of Racemic Epoxide **1-*rac*** through Epoxidation of 1,5-Hexadiene with mCPBA

**Scheme 2:**
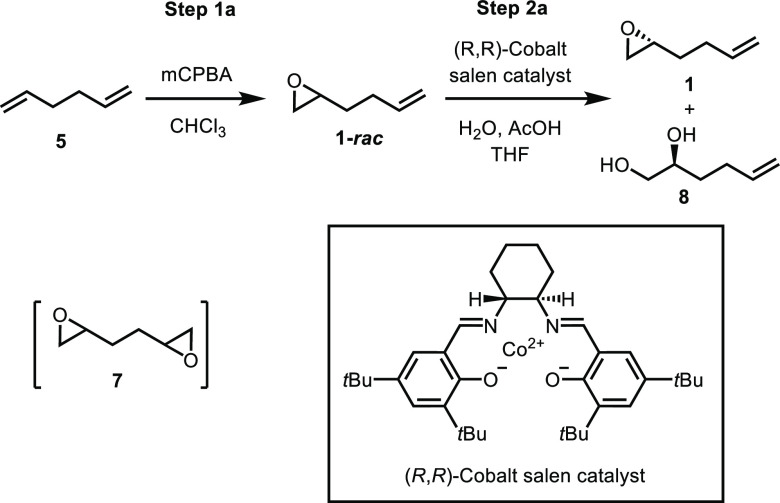
Resolution Route for the Synthesis of Chiral Epoxide **1**

At the outset of our work, the chiral epoxide **1** was prepared in two steps according to the literature method: (1) epoxidation of 1,5-hexadiene **5** with mCPBA to obtain the racemic epoxide **1-*rac***(24,27) and (2) HKR of **1-*rac*** with Jacobsen’s cobalt salen catalyst to afford the chiral epoxide **1** (Scheme 2).^15^ Adding 1,5-hexadiene to mCPBA at 0 °C (reverse addition), the epoxidation was conducted with a stoichiometric amount of mCPBA in CHCl_3_ (25 V) followed by stirring the mixture for 24–30 h (with warming to 25 °C). This conversion is reported to give a 65% isolated yield of the monoepoxide **1-*rac***.^24^ Although there was no mention of bis-epoxide **7** in the original report, we observed a significant amount of bis-epoxide **7** (∼20%) using these conditions. Additionally, the isolated yield of monoepoxide **1-*rac*** was only 41%. The overepoxidation might be attributed to the excess amount of mCPBA during the initial charging of the 1,5-hexadiene with the reverse addition. This two-step protocol utilizes inexpensive starting materials—providing a potentially cost-effective route for the synthesis of the chiral epoxide. However, the current method is not favorable for scale-up due to (1) the formation of a significant amount of undesired bis-epoxide **7** (∼20 A %); (2) the employment of chloroform [a class 2 solvent, with low permitted daily exposure (PDE) (0.6 mg/day) and concentration limit (60 ppm) due to its inherent toxicity].^28^

Based on the aforementioned reasons, a comprehensive optimization of this two-step process was carried out to ensure a viable approach for the synthesis of the chiral epoxide in scale. Our initial solvent optimization identified that dichloromethane (DCM) was the solvent of choice (PDE: 6.0 mg/day, concentration limit: 600 ppm). The reaction in DCM was completed within 3 h, giving the desired epoxide **1-*rac*** in an ∼60 A % of the yield and the bis-epoxide **7** in ∼16 A %. The investigation of the addition order of the reagent showed similar results between the reverse addition (adding 1,5-hexadiene to mCPBA) and the regular addition (adding mCPBA to the 1,5-hexadiene). For operational practicality, the regular addition was used for all of the following investigation. It was observed that warming the reaction to room temperature might not be necessary and could be one of the reasons for the higher amount of bis-epoxide **7** formation. Therefore, the reaction in DCM was performed below 3 °C; however, a similar reaction profile was observed with just a minor decrease in the formation of the bis-epoxide (see Table S1 for details). Although no significant reduction in the bis-epoxide **7** impurity was observed, this optimization informed the desired parameters for the design of experiments (DOE) analysis.

Based on the prior results, a DOE analysis was performed by varying four parameters: (1) molar ratio of mCPBA and 1,5-hexadiene (0.4 to 2.0); (2) solvent volume (5 to 25 V); (3) reaction time (20 to 180 min); and (4) reaction temperature (−11 to 29 °C). According to the DOE analysis, 27 experiments were designed and conducted. The detailed experimental setup and results are presented in Table S3 (see Supporting Information). Parenthetically, identical outcomes for the three center point reactions confirmed the validity of this DOE analysis. For all of these reactions, the desired epoxide **1-*rac*** was monitored by GCMS. As expected, the outcome highly relied on the reaction temperature and the amount of 1,5-hexadiene. For example, at 19 °C, up to 60 A % of unwanted bis-epoxide **7** was observed when 1,5-hexadiene was the limiting reagent. In contrast, less than 10 A % of the unwanted bis-epoxide 7 was observed when performing the reaction at −1 °C (under otherwise a similar reaction condition). An excess of 1,5-hexadiene is critical to minimize the formation of bis-epoxide and achieve high yield of the desired monoepoxide. For instance, at 9 °C, the use of 2.0 equiv of 1,5-hexadiene afforded the bis-epoxide **7** less than 5 A % while **1-*rac*** was up to 92 A %. However, with a molar ratio of 2:1 mCPBA to 1,5-hexadiene, **1-*rac*** is obtained in a 48 A % yield, while the undesired bis-epoxide **7** was up to 45 A % yield.

Based on the initial results from the DOE analysis, further optimization was designed to optimize the solvent volumes of this transformation. With 2.0 equiv of 1,5-hexadiene, two sets of reactions with varied solvent volumes at two different temperatures (0 and 10 °C) were performed (Table S4). It was found that the optimal solvent volume is 5 V and the optimal reaction temperature is about 0 ± 5 °C. Under this optimized condition, **1-*rac*** was obtained >90 A %, and the formation of bis-epoxide **7** was less than 10 A %.

With the optimized process in hand, two batches of reactions with 150 g of 1,5-hexadiene were conducted to prepare epoxide **1-*rac*** in a 2 L ChemRxnHub reactor (Table 1). In the first batch, a mixture of 1,5-hexadiene (150 g) and 5 vol of DCM was cooled to 0 °C, and then 210 g of mCPBA was added portionwise over 50 min. After the addition of the mCPBA, the resulting suspension was stirred at 0 °C for another 3 h, and the mixture was quenched by an aqueous solution of NaOH (2 N). Two clear layers of solution were formed, and the organic phase (in-solution) yield of **1-*rac*** was up to 88 A % and the formation of bis-epoxide **7** was just 8 A %. Crude ^1^H NMR and a peroxide strip test indicated the removal of peroxy materials. The mixture was then subjected to an atmospheric distillation at 50–100 °C, and 32 g of 1,5-hexadiene was recovered as a DCM solution. The resulting crude material was further refined by distillation at 170 °C, and 47 g of epoxide was obtained with a purity of 85 wt % by GCMS. The corrected isolated yield of epoxide was 42%. It should be noted that a drastic amount of product loss occurred during the solvent purge and distillation due to the low boiling point of the epoxide (∼120 °C) and 1,5-hexadiene (∼60 °C). To improve the recovery yield, we decreased the distillation temperature; meanwhile, N_2_ flow was used throughout the distillation process. This operation increased the recovery yield dramatically. As shown in the second batch, the epoxidation gave an in-solution yield of **1-*rac*** up to 95 A % after the aqueous workup. The resulting DCM solution was distilled at 50–80 °C with N_2_ flow (0.1 N L/min), and 71 g of 1,5-hexadiene was recovered along with DCM. The recovery yield of 1,5-hexadiene was 95%. Further distillation at 170 °C with N_2_ flow gave 67 g of epoxide **1-*rac*** with a purity of 95 wt %. The isolated yield of **1-*rac*** was 71%.

**Table 1:**
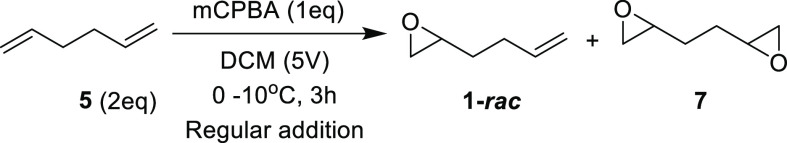
Scale-Up of Epoxidation of 1,5-Hexadiene **5** with mCPBA^a^

entry	scale (g)	temp (°C)	in-solution yield (TIC A %)^b^			isolated yield % (g)^c^		**1-*rac*** purity^f^ wt %
			**1-*rac*** (%)	**7** (%)	**5** (%)	**1-*rac***	**5**	
1	150	0 ± 5	88	8	94	42% (47 g)	43% (32 g)^d^	85%
2	150	0 ± 5	95	5	100	71% (67 g)	95% (71 g)^e^	94%

a See Experimental Section for details.

b In-solution yields were calculated based on GCMS (A %) by total ion chromatogram.

c The corrected isolated yield was calculated based on the wt % purity.

d Atmospheric distillation.

e Atmospheric distillation with a N_2_ flow.

f The wt % purity was obtained from GCMS (wt %) compared to a standard of known purity.

### 2.2 Hydrolytic Kinetic Resolution of **1-*rac*** to Synthesize Chiral Epoxide **1**

With racemic epoxide **1-*rac*** in hand, we sought to develop a scalable process for the resolution of the epoxide following Jacobsen’s HKR procedure to prepare chiral epoxide **1**. Jacobsen’s HKR procedure enriches one enantiomer of the epoxide by enantioselective ring opening, a process catalyzed by a chiral (salen)Co(II) catalyst. In the case of **1-*rac***, the (*R*,*R*)-chiral (salen)Co(II) catalyst promoted the (*S*)-epoxide to allow the ring-opening reaction with water, and as a result, **1-*rac*** was resolved to obtain (*R*)-epoxide **1**.^15^ The Jacobsen’s HKR is well developed and finds a myriad of applications in small-scale chiral epoxide synthesis and utilizes cost-effective reagents.^29,30^ To obtain an optimal catalyst loading for scale, we screened the catalyst loading against the original 0.5 mol % catalyst loading with 1 g-scale reactions. After extensive experimentation, we found that the original 0.5 mol % catalyst loading was optimal (Figure S3). As reported, the experiment with 0.5 mol % of catalyst loading was complete after 16 h, affording compound **1** in up to 99.6% ee. Then the condition was demonstrated on 8–60 g scale reactions. As shown in Table 2, the two smaller-scale reactions performed well; however, the 60 g scale batch proceeded much slower. The reason for the slow reaction on the large scale is unknown, but it suggests challenges of this HKR process for the synthesis of epoxide **1** in scale-up. It should be noted that a significant amount of product was lost during the distillation. A higher recovery yield in distillation for a large-scale production is expected.

**Table 2:**
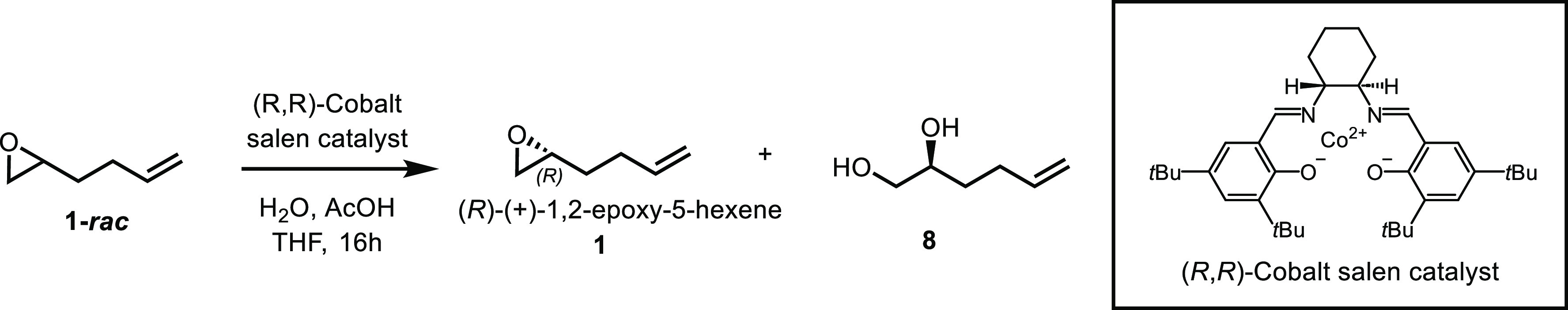
Scale-Up of the Hydrolytic Kinetic Resolution of *rac*-1,2-Epoxy-5-hexene **1-*rac***^a^

entry	scale (g)	ee (%)^b^	assay yield (%)^c^	isolated yield (g)^d^	purity (%)^e^
1	8	95	44	40% (3.2 g)	100
2	20	96	49	33% (6.6 g)	93
3^f^	60	94	49	34% (20.4 g)	99

a All reactions were performed with (salen)Co(II) (0.5 mol %), AcOH (2 mol %), THF (0.1 mL/g), air, water (0.55 equiv), 0 °C-rt, 16 h.

b Monitored by chiral GC.

c Assay yield determined by GCMS.

d Isolated yield determined after distillation under vacuum (90 °C, 90 Torr).

e The purity was obtained by GCMS (A %).

f The ee was achieved after 160 h.

The resolution route (two-step) affords the chiral epoxide **1** from readily available 1,5-hexadiene in up to 25% overall yield in hundreds of gram-scale reactions. This route exhibits promise for scale-up; however, the inherent low yield (no greater than 50%) of the HKR step results in a low overall yield. To obtain a more practical and cost-effective route for the synthesis of chiral epoxide **1**, a route utilizing inexpensive and readily available (*R*)-epichlorohydrin **6** as the starting material was then investigated.

### 2.3 Synthesis of Chiral Epoxide **1** through the Epichlorohydrin Route (Route 2)

#### 2.3.1 Synthesis of Chlorohydrin **10** through Ring Opening of (*R*)-Epichlorohydrin **6**

**Scheme 3:**
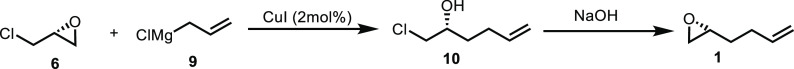
Reported Method to Synthesize Chiral Epoxide **1** from Chiral Epichlorohydrin **6**

We initially evaluated the literature method to make epoxide **1** through the ring-opening reaction of 1.0 equiv of (*R*)-epichlorohydrin **6** with 1.2 equiv of allylMgCl **9**, followed by NaOH treatment (Scheme 3).^22,31−33^ In the presence of 2 mol % CuI, (*R*)-epichlorohydrin **6** reacted with allylMgCl **9** to afford intermediate **10** in an ∼60% yield after column purification. Epoxide **1** was obtained in a quantitative yield by the reaction of intermediate **10** with NaOH pellets. This two-step process shows promise for the synthesis of chiral epoxide **1** from economical starting materials. However, the low yield of the first ring-opening reaction and the need for column purification impede scale-up. The impurity profile of the crude reaction mixture of the ring-opening reaction was investigated by GCMS. It was found that the major impurities included 1,5-hexadiene **5** (up to 20%), 1,8-nonadien-5-ol **11** (up to 2%), and dichlorohydrin **12** (up to 4%). For a scalable process development of the epichlorohydrin route, we aimed to (1) minimize the formation of 1,5-hexadiene **5** and other side products **11** and **12**, thus improving the yield of the compound **10** and (2) eliminate the need for column purification, minimizing processing costs and enabling scalability.

As shown in Table 3, our first attempt was to screen equivalents of allylMgCl **9** in the presence of 2 mol % of CuI. With the equivalents of allylMgCl **9** from 0.8 to 1.2, it was found that a significant amount of 1,5-hexadiene (up to 22 A %) always formed (Table 3, entries 1–3). The side products of 1,8-nonadien-5-ol **11** and dichlorohydrin **12** were also observed under these conditions. Specifically, both **11** and **12** were formed at a level of about 7% when a stoichiometric amount of the Grignard reagent was used (Table 3, entry 2). It is assumed that trace amounts of oxygen promote the homocoupling reaction of the Grignard reagent to form 1,5-hexadiene.^34^ However, a careful removal of oxygen by purging the reaction mixture with argon gave no improvement; in contrast, the reaction proceeded similarly well under air (Table 3, entry 4). Intriguingly, the formation of 1,5-hexadiene **5** was drastically suppressed in the absence of CuI. Without the addition of CuI, the reaction of epichlorohydrin **6** with 1.2 equiv of allylMgCl **9** afforded the compound **10** in up to 81 A % with only 1.1 A % of 1,5-hexadiene (Table 3, entry 5). Notably, under these conditions, 1,8-nonadien-5-ol **11** and dichlorohydrin **12** were not detected. Encouraged by this promising result, we further optimized the conditions without the use of CuI by investigating the addition order, the equivalents of allylMgCl **9**, and the reaction temperature.

**Table 3:**
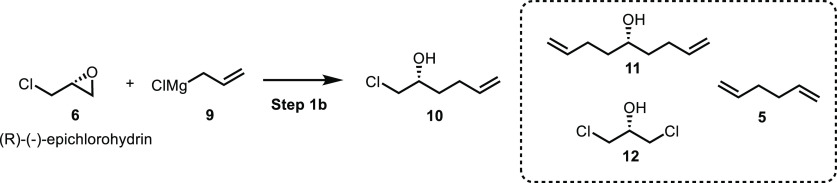
Optimization of Ring Opening of (*R*)-Epichlorohydrin **6** with Allyl MgCl **9**^a^

entry	conditions	**6** A %	**10** A %	**11** A %	**12** A %	**5** A %
1	**6:9** = 1:0.8, regular addition,^b^ 2 mol % CuI, rt, THF (10 V)	9.6	52.7	9.6	9.4	16.8
2	**6:9** = 1:1, regular addition, 2 mol % CuI, rt, THF (10 V)	4.6	58	7.3	6.8	18.5
3	**6:9** = 1:1.2, regular addition, 2 mol % CuI, rt, THF (10 V)	-	60.3	1.6	3.5	21.5
4	**6:9** = 1:1.2, regular addition, 2 mol % CuI, −10°C, THF (10 V), air	-	59.3	5	-	18.5
5	**6:9** = 1:1.2, regular addition, **without CuI**, −10°C, THF (10 V)	-	81	-	-	1.1
6	**6:9** = 1.05:1, **reverse addition**,^c^ without CuI, 10°C, neat	-	93.9	6.0	0.9	-
7	**6:9** = 1.05:1, reverse addition, without CuI, 20°C, neat	-	91.2	0.4	1.4	-
8	**6:9** = 1.05:1, reverse addition, without CuI, 20°C, neat (25 g)	-	73.7	-	9.1	-
9^d^	**6:9** = 1:1, **regular addition**, **without CuI**, −5°C, THF (1 V)	-	94.3	-	2.2	-
10^e^	**6:9** = 1:1, regular addition, without CuI, −5°C, THF (1 V)	-	86.6	-	2.1	-
11^f^	**6:9** = 1:1, regular addition, without CuI, −5°C, THF (1 V)	-	81.7	-	2.1	-

a All reactions were carried out with **6** (1 g) and allylmagnesium chloride (**9**, 2 M in THF) in THF under dry nitrogen at a given molar ratio as shown in the table, 1 h, quenched with MeOH, neutralized by aq HCl (2 M), extracted with EtOAc, and analyzed on GCMS by total ion chromatogram.

b Regular addition: adding the Grignard reagent to the solution of epichlorohydrin in THF dropwise.

c Reverse addition: adding epichlorohydrin to the Grignard reagent dropwise.

d 10 g of **6** was used, quenched by aq 2 M HCl.

e 25 g of **6** was used, quenched by saturated aq NH_4_Cl.

f 25 g of **6** was used, quenched by aq H_2_SO_4_ (1 M).

For a more convenient operation in scale, the addition of epichlorohydrin to the Grignard reagent (reverse addition) was investigated. Without the use of CuI, the reverse addition fashion showed promise on a gram scale. For instance, the reaction of 1.05 equiv of (*R*)-epichlorohydrin **6** with 1.0 equiv of the Grignard reagent **9** at 10 °C afforded the desired product **10** in 94 A %, and the formation of **11** and **12** was only 0.9 and 1.2 A %, respectively (Table 3, entry 6). A similar result was obtained when the internal temperature was kept below 20 °C (Table 3, entry 7). Unfortunately, when we scaled to 25 g of (*R*)-epichlorohydrin **6** under these conditions, up to 9 A % of **12** was generated (Table 3, entry 8), which indicated the competition reaction of the epichlorohydrin with chloride anion at scale. As a result, the regular addition approach (adding the Grignard reagent to the epichlorohydrin) was revisited. The prior results indicated that the use of excess Grignard reagent resulted in the formation of both side-products **11** and **12**, while the excess (*R*)-epichlorohydrin **6** underwent ring-opening reaction with a chloride anion to form the side-product **12** as well. After fine-tuning the ratio of the epichlorohydrin and Grignard reagent, it was found that the stoichiometric reaction of the epichlorohydrin and Grignard reagent afforded the best results (Table 3, entries 9–11). Under a regular addition mode, the optimal internal temperature was identified as 0 ± 5 °C, and the optimal volume of the solvent was found to be 1 V. As a result, with a regular addition mode, the stoichiometric reaction between the allylMgCl **9** and (*R*)-epichlorohydrin **6** at 0 ± 5 °C in 1 V of THF afforded the desired product **10** in 94 A %. The side product of **11** was not detected, and the formation of **12** was only 2.2 A %. Notably, the side product of 1,5-hexadiene **5** was also not detected under this condition (Table 3, entry 9). This process showed good repeatability as comparable results were obtained in a 25 g scale reaction. It should be noted that the MeOH quench is critical before the workup.^31^ A clear aqueous and organic phase separation was achieved with a MeOH quench followed by an aq HCl (2 M) neutralization. However, without the MeOH treatment, a direct acid neutralization of the reaction mixture generated a gel-like mass, precluding further purification. Notably, a similar impurity profile was obtained when the reaction mixture was acidified with aq H_2_SO_4_ (1 M) or saturated aq NH_4_Cl (Table 3, entries 10 and 11). In all cases, the aqueous layer contained less than 5 A % product by GCMS after a one-time MTBE extraction. Attempts to separate compound **10** from the side-products **11** and **12** by distillation failed due to a similar boiling point of these compounds.

#### 2.3.2 Synthesis of Chiral Epoxide **1** through Ring Closure of Chlorohydrin **10**

A previous report described the NaOH-promoted conversion of the chlorohydrin **10** to epoxide **1**, although this was done on a small scale.^22^ To our delight, the ring closure reaction of crude **10** went smoothly in the presence of 2.0 equiv of NaOH pellets at 60 °C, and up to 94 A % yield of epoxide **1** was obtained. Impurity **12** was not detected, but epichlorohydrin **6-*rac*** was observed in 1.5 A %, indicating that compound **12** underwent ring closure as well (Table 4, entry 1).

**Table 4:**
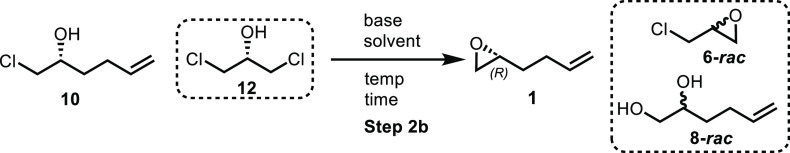
Optimization of Ring Closure of **10** with Base^a^

entry	conditions	**1** (A %)	% ee	**6-*rac*** (A %)
1	NaOH (2 equiv, pellets), neat, rt −60°C, 2 h	94	99.9	1.5
2	K_2_CO_3_ (2 equiv), neat, 60°C, 2 h	44^b^	-	-
3	K_2_CO_3_ (1 equiv), ethylene glycol, 60°C, 4 h	88^b^^,^^c^	-	-
4	DIPEA (1 equiv), ethylene glycol, 60°C, 4 h	0^b^	-	-
5	DIPEA (1 equiv), diglyme, 60°C, 4 h	0^b^	-	-
6	NaOH (2 equiv, 2 M), water, rt, 12 h	72.8^d^	-	-
7	NaOH (1.2 equiv, 2 M), MTBE (2 V), rt, 12 h	95	99.9	2
8	NaOH (1.2 equiv, 2 M), MTBE (2 V), 50°C, 2 h	98^e^	99.9	2

a All reactions were performed with **10** (1 g, 1 equiv) and base under the conditions as indicated in the table, and **8-*rac*** was not observed in all conditions; A % was calculated based on GCMS by total ion chromatogram.

b Estimated by ^1^HNMR.

c Ring opening with ethylene glycol was the major side-reaction.

d 20.6 A % of **10** was unreacted.

e About 1.5 A % of **10** remained.

Although NaOH showed great promise, the use of solid NaOH is not amenable to scale-up. Additionally, it is noted that utilizing NaOH at 60 °C may cause etching of glass reactors. To develop a more practical process for the scale-up of the ring closure reaction, we screened different bases, solvents, and temperatures. As summarized in Table 4, a milder base, K_2_CO_3_, under a similar condition, gave a poor conversion with only 44 A % of **1** (Table 4, entry 2). In a high boiling point solvent such as ethylene glycol, the reaction with K_2_CO_3_ proceeded more efficiently. For instance, the treatment of the crude **10** with K_2_CO_3_ in ethylene glycol afforded the epoxide in 88 A %; unfortunately, about 10 A % of adduct of the epoxide with ethylene glycol was also observed (Table 4, entry 3). The use of DIPEA gave no product in either ethylene glycol or diglyme at 60 °C (Table 4, entries 4 and 5). As a result, NaOH was revisited for the epoxide formation. Initial results showed that an aqueous solution of NaOH (2 N) was promising for the ring closure of compound **10**. Treating chlorohydrin **10** with 2.0 equiv of NaOH (2 N) at room temperature, the desired epoxide was obtained in a 73 A % yield after 12 h, and about 21 A % starting alcohol remained (Table 4, entry 6). When switching the solvent to the MTBE, 1.2 equiv of NaOH (2 N) enabled a full conversion within 12 h at room temperature (Table 4, entry 7). Notably, the hex-5-ene-1,2-diol (**8-*rac***) was not detected under these conditions, which indicated the chemical inertness of epoxide **1** with NaOH. Increasing the reaction temperature to 50 °C was sufficient to afford a satisfactory conversion (>98%) within 2 h. For instance, the treatment of compound **10** with 1.2 equiv of NaOH (2 N) in MTBE (2 V) at 50 °C for 2 h produced epoxide **1** in 98 A % conversion (Table 4, entry 8). The successful epoxidation in MTBE enabled a possible direct use of the resulting MTBE solution from the prior ring-opening reaction without the need for solvent swap. When treating the MTBE solution of the crude product **10** (from step 1b) with 1.2 equiv of NaOH (2 N) at 50 °C for 2 h, full conversion was obtained, the in-solution yield was up to 90 A %, and the purity was up to 96 A % after water wash. It is worth mentioning that all epoxide obtained through this process was the (*R*)-enantiomer (>99.9% ee), as confirmed by a chiral GC analysis.

### 2.4 Scale-Up of the Epichlorohydrin Route (Route 2) for the Synthesis of Chiral Epoxide **1**

With the optimized two-step epichlorohydrin route for the production of **1** in hand, the scale-up of the process in a 100 g scale (100–200 g) was demonstrated in a 5 L ChemRxnHub reactor (Table 5). This process generated the epoxide with an overall in-solution yield of 70–77 A %, a chemical purity of up to 96 A %, and an enantiomeric excess of up to 99.9%. After distillation in the second step, 56–60% isolated yield and up to 99 A % purity were achieved. For instance, as shown in entry 1 (Table 5), at the scale of 150 g of the epichlorohydrin **6**, its stoichiometric reaction with the allylMgCl **9** afforded the chlorinated alcohol **10** in 93 A % in-solution yield after workup [quenched by MeOH, neutralized with aq HCl, and extracted by MBTE (750 mL)]. The major impurity was dichlorohydrin **12** (2.5 A %). The crude **10** in MTBE underwent the ring closure reaction smoothly by treating with 1.2 equiv of NaOH (2 N) at 50 °C for 2 h. After washing the organic phase with water until the aqueous phase showed pH = 7, the resulting MTBE solution of **1** exhibited an in-solution yield of 70 %. As was found on the small scale, compound **12** was not observed, but epichlorohydrin **6-*rac*** was detected (1 A % yield). After removal of the MTBE, the resulting crude product was purified by vacuum distillation (70–80 Torr at 80 °C), affording compound **1** in a 56–60% isolated yield with excellent chemical and enantiomeric excess purities (Table 5, entries 1 and 4). It should be noted that up to 20 % of compound **1** was lost during the solvent purge and distillation. Other distillation approaches, such as normal pressure distillation w/o N_2_ purge, resulted in a similar recovery yield (Table 5, entries 5 and 6).

**Table 5:**
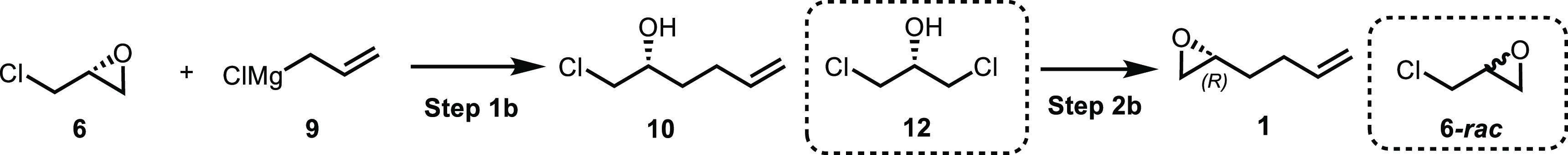
Two-Step Process for the Synthesis of Epoxide **1** from (*R*)-Epichlorohydrin **6**^a^

step 1b: ring opening of epichlorohydrin to form **10**			
entry	scale (g)	compound **10** (in-solution yield) (%)^b^	impurity **12** (%)^c^
1	150	93	2.5
2	150	94	3.9
3	200	91	4.0

step 2b: ring closure of **10** to yield epoxide **1**					
entry	scale (g)	in-solution yield of **1** (%)	in-solution purity^c^		yield of **1** after distillation^d^
			epoxide **1** (%)	impurity **6-*rac*** (%)	gram, yield %, A % purity, ee^e^
4^f^	-	70	96	1.0	97 g, 60%, 99 A %, 99.9%
5^g^	-	76	96	1.2	91 g, 56%, 98 A %, 99.9%
6^h^	-	77	98	1.5	125 g, 59%, 96 A %, 99.9%

a See Experimental Section for details.

b All the in-solution yields were calculated based on GCMS (wt %).

c The impurity percentage was obtained by GCMS (A %).

d 0.4 A % of 1,8-nonadien-5-ol **11** was also detected. The area purity was obtained by GCMS (A %), and the mass/yield was corrected data.

e The ee was obtained by chiral column GC.

f The distillation of compound **1** was performed under 70–80 Torr at 80 °C.

g Atmospheric distillation at 170 °C with N_2_ flow.

h Atmospheric distillation at 170 °C without N_2_ flow.

## 3 Conclusions

In conclusion, two scalable routes for the synthesis of *R*-(+)-1,2-epoxy-5-hexene **1** were developed from inexpensive, readily available starting materials. Route 1 involves epoxidation and the Jacobsen HKR. The epoxidation of 2 equiv of 1,5-hexadiene with 1 equiv of mCPBA afforded the assay yields of up to 95 A %. The following resolution step achieved the assay yield up to 49 A % (vs theoretical yield: 50 A %). The two-step process was demonstrated on a 100 g scale, affording the chiral epoxide **1** with an overall yield of ∼25% after distillation. It is worth noting that although the HKR can only generate maximum 50% yield, it is distinctive of method 2 in that no chiral pool material is required.

Route 2 employed (*R*)-epichlorohydrin as the starting material and included an epoxide ring-opening reaction and a ring closure reaction. Development of this process successfully minimized the formation of side products, thereby enabling the formation of the enantiopure epoxide **1** with an overall isolated yield of up to 56–60% and purity of up to 99 A % on a hundred-gram scale. Additionally, the excellent purity profile of crude epoxide **1** from the epichlorohydrin route provides possible telescoping options to subsequent synthetic steps. For example, this allows implementation of the Hodgson reaction to produce **2**. Compared to the resolution route, the epichlorohydrin route provides a more efficient and scalable strategy to prepare this chiral building block, (*R*)-1,2-epoxyhex-5-ene (**1**).

We hope that these scalable approaches to the chiral epoxide **1** will inspire more applications of its use in organic synthesis routes, including further efforts to optimize the process toward the cost-effective synthesis of lenacapavir.

## 4 Experimental Section

### 4.1 General Information

Reagents and solvents were obtained from commercial suppliers and used as received unless otherwise indicated. (*R*)-Epichlorohydrin (99%) was purchased from Oakwood Chemical, and allylmagnesium chloride solution (2.0 M in THF) was purchased from Sigma-Aldrich. Reactions were conducted in oven-dried (120 °C) glassware, which was assembled while hot and cooled to ambient temperature under an inert atmosphere. All reactions were conducted under an inert atmosphere (N_2_) unless otherwise noted. Reactions were monitored by TLC (precoated silica gel 60 F254 plates, EMD Chemicals), GCMS, or chiral-column GC using various methods. TLC was visualized with UV light or by treatment with phosphatidyl alcohol (PMA), ninhydrin, and/or KMnO_4_. Flash chromatography was performed on a Teledyne ISCO Combi-Flash NEXTGEN 300+ and/or a Biotage Isolera using solvents as indicated. HRMS was recorded using PerkinElmer Axion 2 ToF MS, ionization mode: positive with scan range: 100–1000 *m*/*z*, flight tube voltage: 8 kV, spray voltage: 3.5 kV, solvent: methanol. ^1^HNMR and ^13^CNMR spectra were routinely recorded on a Bruker Avance III HD Ascend 600 MHz spectrometer. The NMR solvents used were CDCl_3_ or CD_3_CN as indicated. Tetramethylsilane (TMS) was used as an internal standard. Coupling constants J are reported in hertz (Hz). The following abbreviations were used to designate signal multiplicity: s, singlet; d, doublet; t, triplet; q, quartet, p, pentet; dd, doublet of doublets; ddd, doublet of doublet of doublets; dt, double of triplets; ddt, doublet of doublet of triplets; m, multiplet; br, broad. 1,3,5-Trimethoxybenzene and triphenylmethane were used as internal standards for quantitative ^1^H NMR.

#### 4.1.1 Synthesis of *rac*-(±)-1,2-Epoxy-5-hexene (**1-*rac***) (Step 1a, Resolution Route)

A 2 L ChemRxnHub reactor was charged with 750 mL of DCM (5 V) and 1,5-hexadiene (150 g, 1.83 mol, 2 equiv), and the reaction solution was cooled to −5 °C (internal temperature −3.8 °C) with a chiller. Solid mCPBA (210.0 g, 912.7 mmol, 1 equiv) was added in three equal portions (3 × 70.0 g), maintaining the internal temperature <5 °C. Once the reaction cooled back down to −3 °C after the final addition, the reaction was assayed for unconsumed mCPBA: ca. 10% mCPBA remained (^1^H NMR, CD_3_CN). The reaction was warmed to 5 °C and stirred for 1 h to complete. At this point, the reaction was quenched with aq NaOH (440 mL, 2.5 N, 0.6 equiv), stirred briefly and separated, and the organic phase was assayed for product epoxide (86.83 g, 97%). The epoxide solution was concentrated to ca. 250 mL at 65 °C, and further distillation of the volatiles continued at 50–80 °C under gentle N_2_ stream (0.1 NL/min) on a separate distillation setup with a 10″ Vigreux column, long-path condenser into a cooled (−78 °C) receiving flask to recover 1,5-hexadiene (71 g, yield: 95%) as a solution in DCM. Once the volatiles were purged, atmospheric distillation continued at 170–200 °C to yield *rac*-1,2-epoxy-5-hexene **1-*rac*** (67.5 g, 93.8 wt %, yield: 71%).

^1^H NMR (600 MHz, CDCl_3_): δ 5.82–5.75 (m, 1H), 5.01 (dq, *J* = 17.1, 1.7 Hz, 1H), 4.93 (dq, *J* = 10.2, 1.7 Hz, 1H), 2.87–2.86 (m, 1H), 2.69–2.68 (m, 1H), 2.42–2.41 (m, 1H), 2.18–2.11 (m, 2H), 1.62–1.53 (m, 2H). ^13^C NMR (150 MHz, CDCl_3_): δ 137.6, 115.1, 51.8, 47.1, 31.8, 30.2. MS-EI (*m*/*z*): (M^+^), 98.1.

#### 4.1.2 Synthesis of *R*-(+)-1,2-Epoxy-5-hexene (**1**) (Step 2a, Resolution Route)

A 250 mL flask with overhead stirring was charged with (*R*,*R*)-(salen)Co(II) (1.82 g, 3.01 mmol, 0.005 equiv). The catalyst was treated with *rac*-1,2-epoxy-5-hexene **1-*rac*** (63.1 g, 93.8 wt %, 602.8 mmol), AcOH (0.69 mL, 12.06 mmol, 0.02 equiv), and 6 mL of THF under aerobic conditions. The reaction flask was cooled to 0 °C, and H_2_O (6.0 mL, 332 mmol, 0.55 equiv) was added in one portion. The reaction was allowed to warm to room temperature and monitored by chiral GC. After stirring for 160 h, the ee was 94%, with an assay yield of 49 A %. At this time, the volatile materials were distilled at 90 °C under a gentle N_2_ stream (0.1 N L/min), followed by vacuum transfer under 90 Torr at 90 °C to afford (*R*)-1,2-epoxy-5-hexene **1** (19.82 g, 602.8 mmol, 33.5%).

^1^H NMR (600 MHz, CDCl_3_): δ 5.82–5.75 (m, 1H), 5.01 (dq, *J* = 17.1, 1.7 Hz, 1H), 4.93 (dq, *J* = 10.2, 1.7 Hz, 1H), 2.87–2.86 (m, 1H), 2.69–2.68 (m, 1H), 2.42–2.41 (m, 1H), 2.18–2.11 (m, 2H), 1.62–1.53 (m, 2H). ^13^C NMR (150 MHz, CDCl_3_): δ 137.6, 115.1, 51.8, 47.1, 31.8, 30.2. MS-EI (*m*/*z*): (M^+^), 98.1.

#### 4.1.3 Synthesis of (*R*)-1-Chlorohex-5-en-2-ol (**10**) (Step 1b, Epichlorohydrin Route)

To a 5 L ChemRxnHub reactor under a nitrogen atmosphere was added THF (200 mL, 1 V) followed by (*R*)-epichlorohydrin **6** (200 g, 2.16 mol, 1 equiv). This mixture was cooled at −25 °C (internal temperature was −15.5 °C) using a chiller. When the internal temperature reached −15 °C, allylmagnesium chloride **9** (1.08 L, 2.16 mol, 1 equiv, 2 M in THF) was added using a peristaltic pump with a flow rate of 5–10 mL/min, maintaining the internal temperature below −5.0 °C. After addition, this mixture was stirred at the same temperature for an additional 1 h. Then methanol (219 mL, 5.4 mol, 2.5 equiv) was added dropwise, keeping the internal temperature below 0 °C, followed by the addition of HCl (2.16 L, 2 M, 2.0 equiv) at 0 °C. The circulating cooling system was turned off, and MTBE (1 L) was added. The organic layer was collected and washed with HCl (400 mL, 2 M) and water (400 mL), respectively. This resulting organic layer (1.8 L) gave an in-solution yield of 91% **10** assayed by GCMS, containing 4% dichlorohydrin **12** and 0.4% 1,8-nonadien-5-ol **11**. The crude compound **10** was used for the next step without further purification. A small amount of pure compound **10** was obtained by column chromatography for the analytical data.

^1^H NMR (600 MHz, CDCl_3_): δ 5.85–5.78 (m, 1H), 5.03 (dq, *J* = 17.1, 1.7 Hz, 1H), 4.96 (dq, *J* = 10.2, 1.7 Hz, 1H), 2.92–2.89 (m, 1H), 2.73–2.72 (m, 1H), 2.46–2.45 (m, 1H), 2.24–2.14 (m, 2H), 1.95 (br, 1H), 1.65–1.56 (m, 2H). ^13^C NMR (150 MHz, CDCl_3_): δ 137.7, 115.3, 70.8, 50.3, 33.3, 29.7. MS-EI (*m*/*z*): (M^+^), 134.1.

#### 4.1.4 Synthesis of *R*-(+)-1,2-Epoxy-5-hexene (**1**) (Step 2b, Epichlorohydrin Route)

A 5 L ChemRxnHub reactor was charged with a solution of chlorohydrin **10** in MTBE (1.8 L). To the reactor was added an aqueous solution of NaOH (1.3 L, 1.2 equiv, 2 N). The mixture was heated to 50 °C and stirred for 2 h. After completion, the organic layer was collected and washed with water (500 mL × 4) until the aqueous-phase reached pH = 7. The resulting organic phase gave an in-solution yield of 77 A % assayed by GCMS (TIC), containing chiral epoxide **1** of 98.6 A % and epichlorohydrin **6-*rac*** of 1.5 A %. The solution was evaporated at 90 °C to remove solvents of MTBE and THF. The resulting crude product was distilled with Vigreux column at 130–170 °C to afford the desired epoxide **1** (125 g, yield: 59%, purity: 99 A % by GCMS, ee: 99.9%).

^1^H NMR (600 MHz, CDCl_3_): δ 5.82–5.75 (m, 1H), 5.01 (dq, *J* = 17.1, 1.7 Hz, 1H), 4.93 (dq, *J* = 10.2, 1.7 Hz, 1H), 2.87–2.86 (m, 1H), 2.69–2.68 (m, 1H), 2.42–2.41 (m, 1H), 2.18–2.11 (m, 2H), 1.62–1.53 (m, 2H). ^13^C NMR (150 MHz, CDCl_3_): δ 137.6, 115.1, 51.8, 47.1, 31.8, 30.2. MS-EI (*m*/*z*): (M^+^), 98.1.

## Supporting Information Available

Additional experimental details and analytical methods (PDF)
