# Supplementary material for: Synthetic Process Development of (R)-(+)-1,2-Epoxy-5-hexene: An Important Chiral Building Block
Source: Org Process Res Dev. 2024 Aug 1;28(8):3197–205. doi: 10.1021/acs.oprd.4c00101 (PMC11334172; doi:10.1021/acs.oprd.4c00101)
Supplement: Supplementary file 1 — op4c00101_si_001.pdf [file op4c00101_si_001.pdf]

Synthetic process development of *R*-(+)-1,2-epoxy-5-hexene: an important chiral building block

*Daryl Guthrie,‡ John M. Saathoff,‡ Rajkumar Lalji Sahani,‡ Aline Nunes De Souza, ‡ Daniel W. Cook, Samuel R. Hochstetler, Justina M. Burns, Roudabeh Sadat Moazeni-Pourasil, Janie Wierzbicki, Saeed Ahmad, Michael G. Laidlaw, B. Frank Gupton, Charles S. Shanahan, Douglas Klumpp, Limei Jin**

Medicines for All Institute, Virginia Commonwealth University, Richmond, VA, 23284-3068.

‡These authors contributed equally.

*Corresponding author: Email: jinl3@vcu.edu

GENERAL METHODS

Reagents and solvents were obtained from commercial suppliers and used as received unless otherwise indicated. Where applicable, reactions were conducted in oven-dried (120°C) glassware, which was assembled while hot, and cooled to ambient temperature under an inert atmosphere. Reactors were pre-rinsed with reaction solvent and subjected to evacuation/back-fill cycles (3x) as necessary. All reactions were conducted under an inert atmosphere (N_2) unless otherwise noted. Reactions were monitored by TLC (precoated silica gel 60 F254 plates, EMD Chemicals), Agilent GCMS or chiral Agilent GC-FID using various methods. TLC was visualized with UV light or by treatment with Phosphomolybdic acid (PMA), ninhydrin, and/or $KMnO_4$. ^1H NMR and ^{13}C NMR spectra were routinely recorded on Bruker Avance III HD Ascend 600 MHz spectrometer. All chemical shifts are reported in parts per million (ppm) relative to residual $CHCl_3$ (7.26 ppm for ^1H, 77.16 ppm for ^{13}C) or tetramethylsilane (0.0 ppm for ^1H and ^{13}C). Coupling constants J are reported in hertz (Hz). The following abbreviations were used to designate signal multiplicity: s, singlet; d, doublet; t, triplet; q, quartet, p, pentet; dd, doublet of doublets; ddd, doublet of doublet of doublets; dt, double of triplets; ddt, doublet of doublet of triplets; m, multiplet; br, broad.

GC-MS Qualitative Methods. Formation of (*R*)-1,2-epoxyhex-5-ene (**1**), via Route 1 and Route 2 was monitored via GC-MS (Agilent 6890 GC-5977 MSD). The column used was an Agilent J&W HP-1 GC Column, 30 m, 0.32 mm, 5.00 µm. The inlet was set to 250 ˚C. A split ratio of 50:1 was used with an injection volume of 1.0 µL. The column flow rate was 1.4 mL/min with GD10 helium as the carrier gas and an inlet pressure of 7.87 psi. The oven was initially set to 60 ˚C for 3 minutes, linearly ramped to 200 ˚C at 12 ˚C/min and held for an additional 4 minutes.

Structures & IDs:

Epoxide formation:

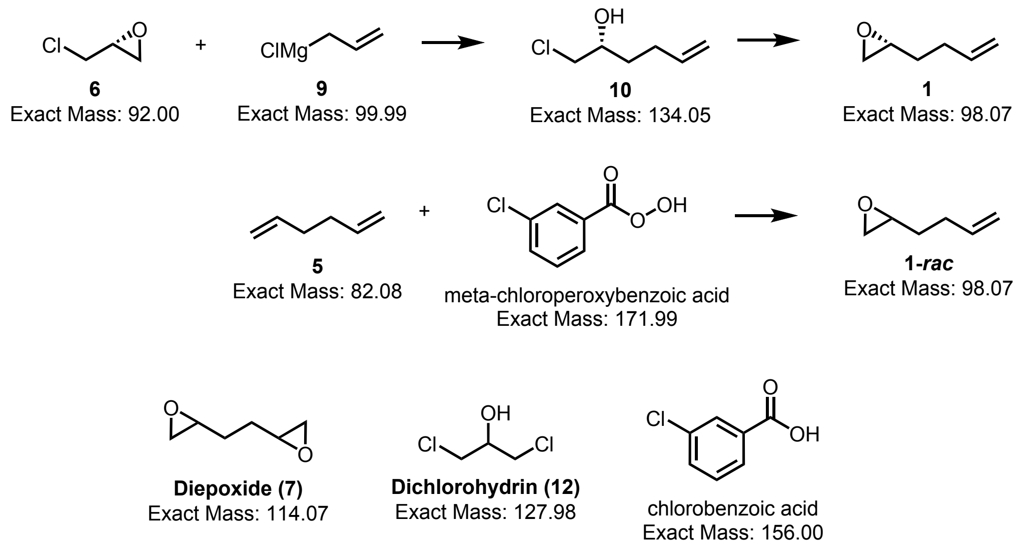

Instrument Type: Agilent 6890 gas chromatograph (GC) with a 5977 mass spectrometer detector (MSD)

Conditions:

Column: J&W HP-1 GC Column, 30 m, 0.32 mm, 5.00 μm, 5 inch cage

Inlet Pressure: 7.87 psi	Split Ratio: 50 : 1	Split Flow: 70 mL/min
Column flow: 1.4 mL/min	Injection Temp: 250 °C	Injection volume: 1 μL
Total Flow: 74.11 mL/min	Solvent Delay: 3.5 min	Runtime: 15 min

Temperature Program:

Time (min)	Ramp (˚C/min)	Temp (˚C)	Hold (min)
Initial	-	60	3
-	12	200	4

MS Parameters:

Transfer Line Temp (˚C)	250
Source Temp (˚C)	230
Quad Temp (˚C)	150
Electron Energy (eV)	70
Mass Range	30-1000

Sample preparation:

Prepare solutions at approximately 0.5 - 1 mg/mL in acetonitrile or methanol for qualitative analysis.

Retention Times

**Compound**	***m/z***	**Time (min)**
MTBE	73	4.36
Hexa-1,5-diene (**5**)	81, 67	4.55
Tetrahydrofuran	72	5.23
Epichlorohydrin **(6)**	62, 57	6.35
*Hexenone**	98, 83	7.05
Monoexpoxide **(1)**	97, 67	7.35
Dichlorohydrin **(12)**	79	8.33
**10**	116, 81	9.19
Diepoxide **(7)**	114, 83	9.10

**Compound assignment based on NIST 17 Mass Spectral Library search*

Notes: na

Representative Chromatogram(s) (attach additional chromatograms and spectra as needed)

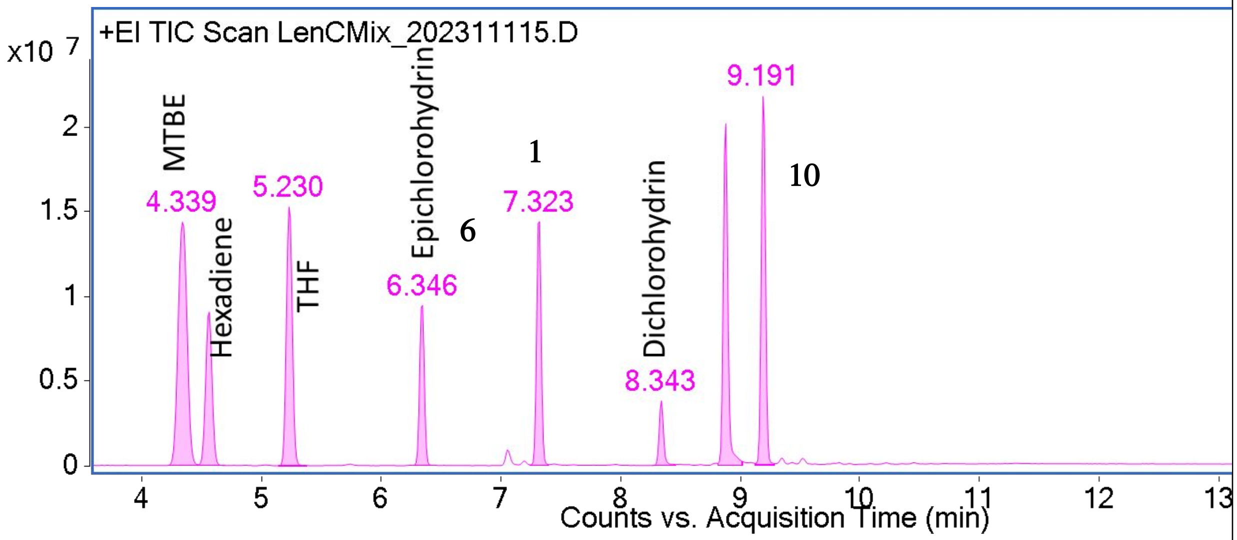

Mass spectra:

MTBE

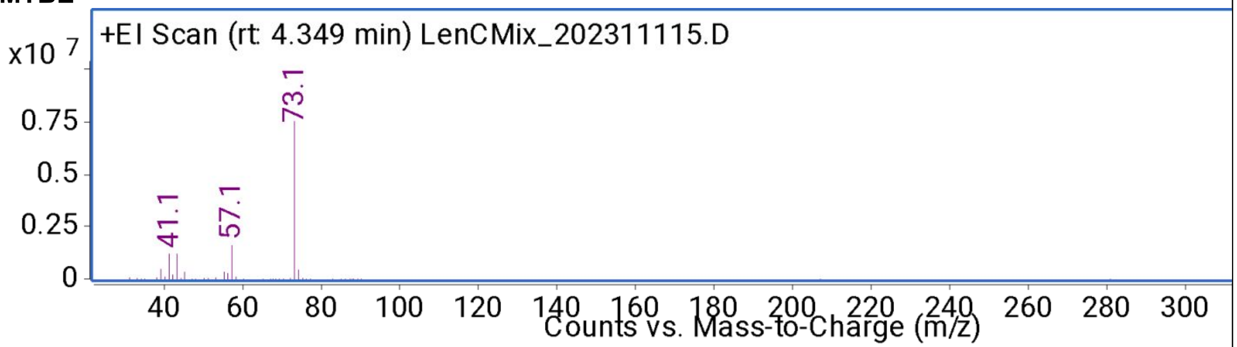

Hexa-1,5-diene

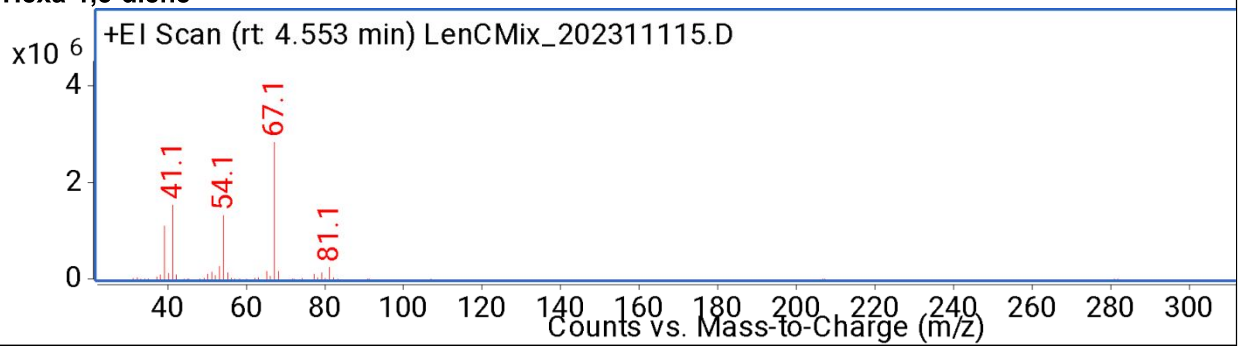

Tetrahydrofuran

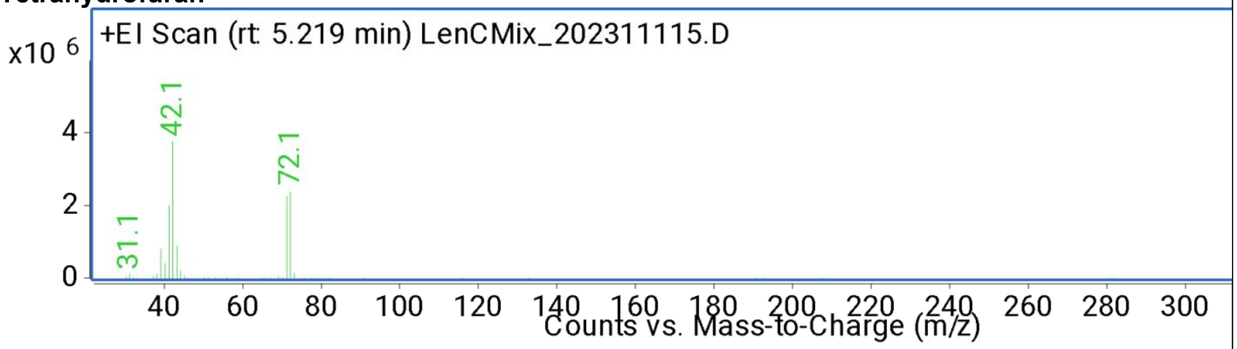

Epichlorohydrin **(6)**

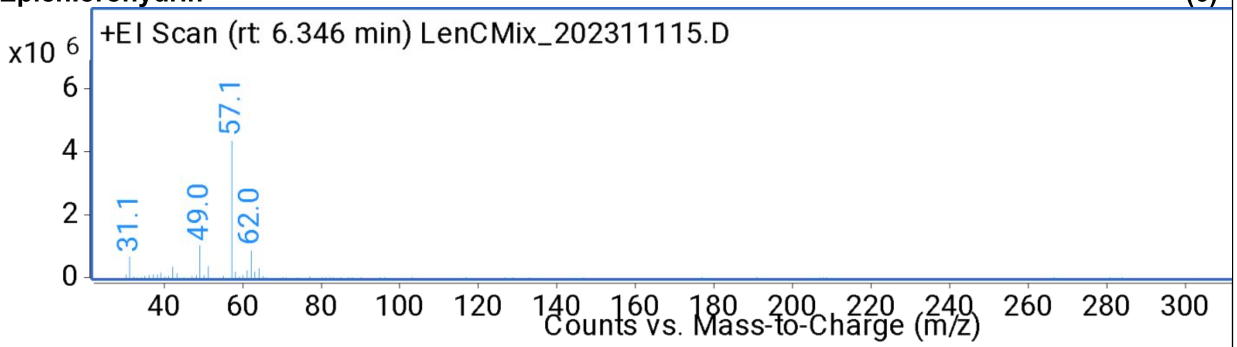

Hexenone*

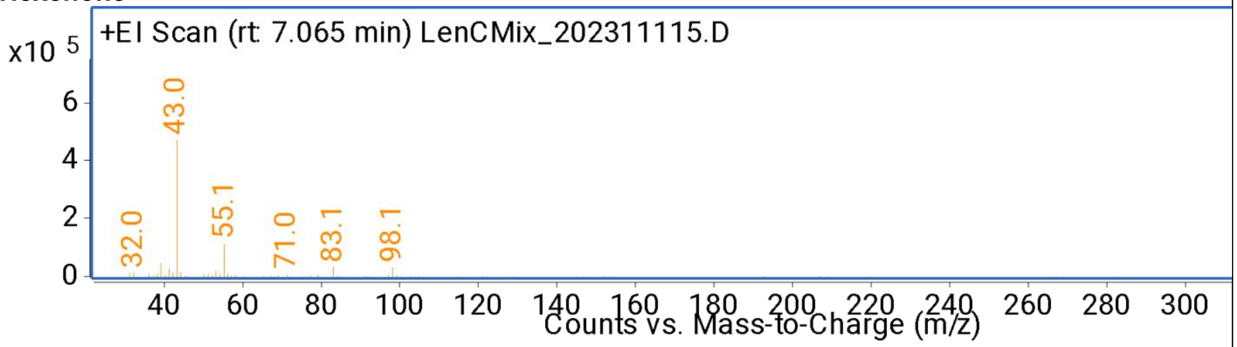

Monoepoxide (1)

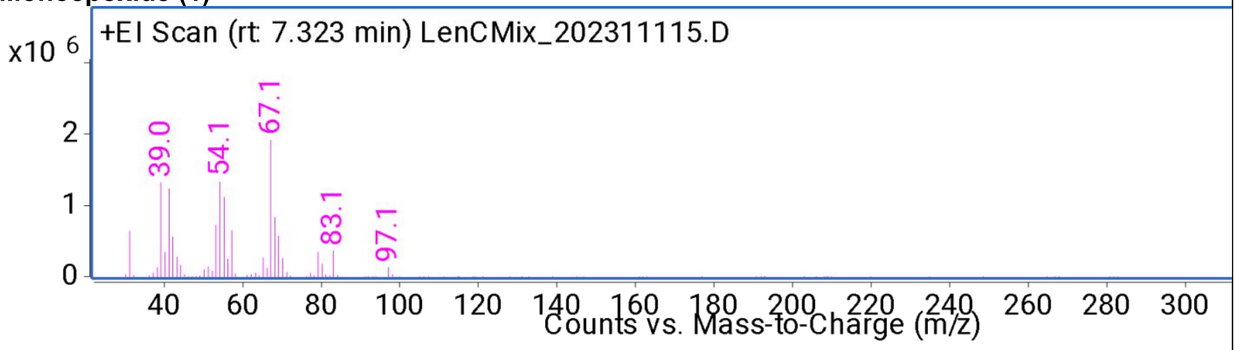

Dichlorohydrin (12)

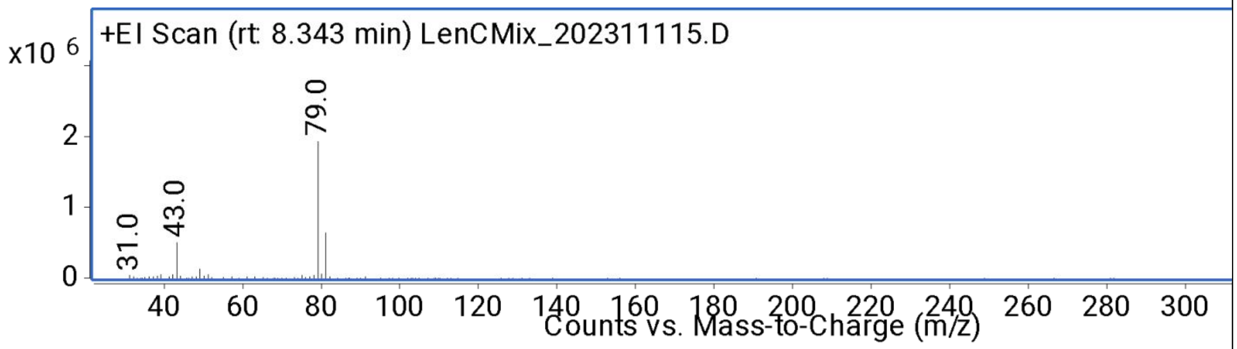

Chlorohydrin (10)

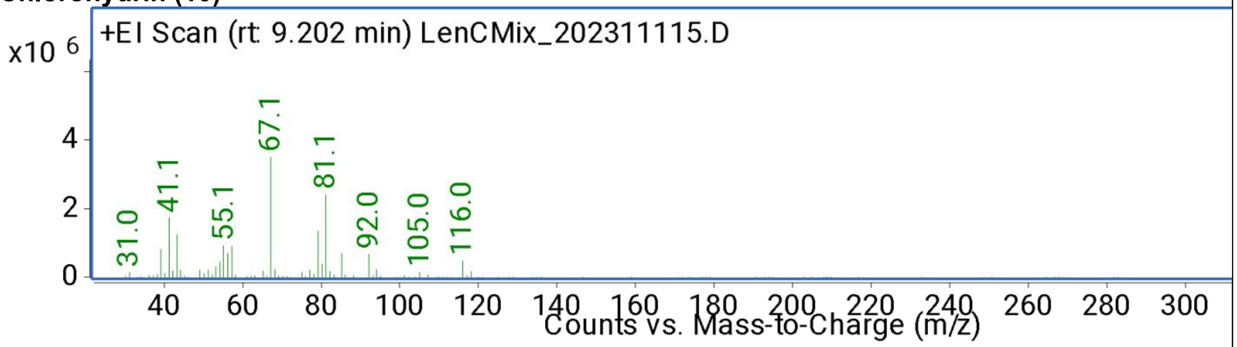

Diepoxide (7)

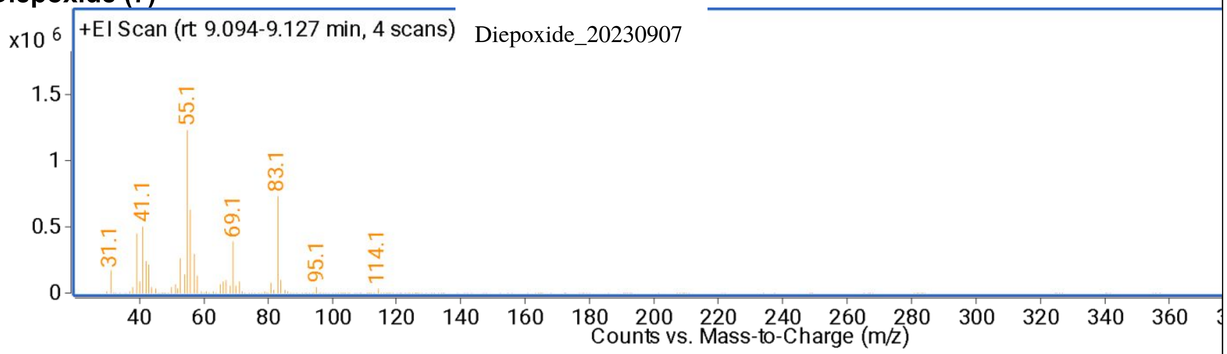

GC-MS Quantitative Methods. Formation of (*R*)-1,2-epoxyhex-5-ene (**1**), via Route 1 and Route 2 was monitored via GC-MS (Agilent 8890 GC-5977 MSD). The column used was an Agilent J&W HP-5ms GC Column, 30 m, 0.25 mm, 0.25 µm, 7-inch cage. The inlet was set to 250 ˚C. A split ratio of 50:1 was used with an injection volume of 1.0 µL. The column flow rate was 1.4 mL/min with helium as the carrier gas and an inlet pressure of 12.4 psi. The oven was initially set to 60 ˚C for 3 minutes, linearly ramped to 200 ˚C at 12 ˚C/min. The *m/z* of 67.0 was extracted from the chromatogram for quantitation.

Structures & IDs:

Epoxide formation:

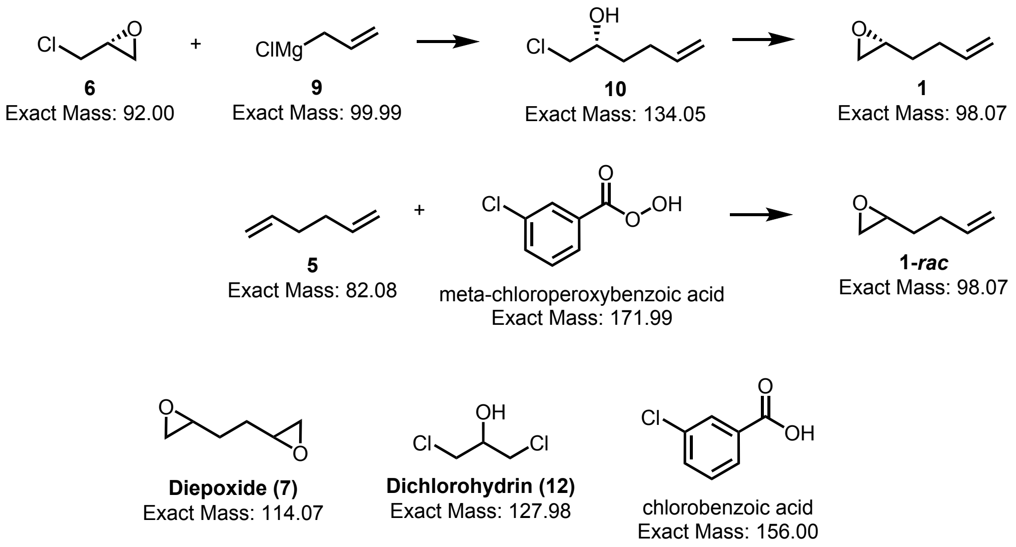

Instrument Type: Agilent 8890 gas chromatograph (GC) with a 5977 mass spectrometer detector (MSD)

Conditions:

Column: J&W HP-5ms GC Column, 30 m, 0.25 mm, 0.25 µm, 7 inch cage

Inlet Pressure: 12.4 psi | Split Ratio: 50 : 1 | Split Flow: 70 mL/min

Column flow: 1.4 mL/min | Injection Temp: 250 ˚C | Injection volume: 1 µL

Total Flow: 74.4 mL/min | Solvent Delay: 2 min | Runtime: 8.4 min

Temperature Program:

Time (min)	Ramp (˚C/min)	Temp (˚C)	Hold (min)
Initial	-	60	3
-	12	200	-

MS Parameters:

Transfer Line Temp (˚C)	250
Source Temp (˚C)	230
Quad Temp (˚C)	150
Electron Energy (eV)	70
Mass Range	40-1000

Chromatogram Processing Parameters:

Extracted Ion Chromatogram (EIC)	67.0

Sample preparation: Prepare solutions at approximately 0.2 – 0.5 mg/mL in acetonitrile or methanol for quantitative analysis. The hexadiene and epoxide (**1**) are quite volatile and must be prepared carefully for quantitation.

Retention Times

**Compound**	***m/z***	**Time (min)**
Monoepoxide **(1)**	97, 67	2.76

Notes: na

Representative Chromatogram(s) (attach additional chromatograms and spectra as needed)

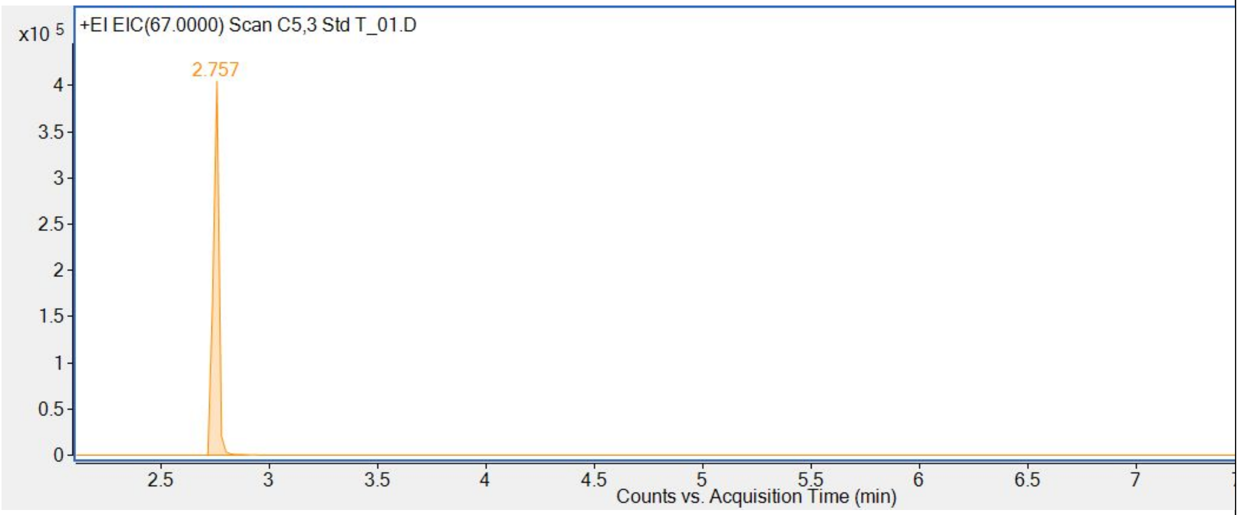

Mass spectra:

Monoepoxide (1)

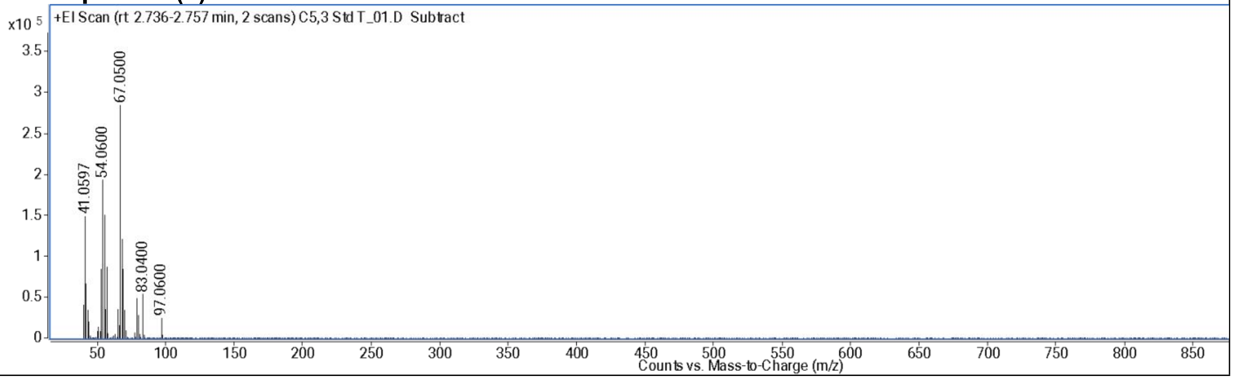

GC-FID Chiral Methods. Formation of (*R*)-1,2-epoxyhex-5-ene (**1**), via Route 1 and Route 2 was monitored via GC-MS (Agilent 6890 GC-FID). The column used was a Restek RT-GammaDEXsa (30 m x 0.25 mm x 0.25 µm). The inlet was set to 250 °C. A split ratio of 50:1 was used with an injection volume of 1.0 µL. The column flow rate was 1.4 mL/min with helium as the carrier gas and an inlet pressure of 16.4 psi. The oven was initially set to 60 °C and held for an additional 16 minutes.

Structures & IDs:

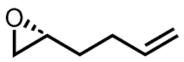

(R)-2-(but-3-en-1-yl)oxirane
Exact Mass: 98.07
(1)

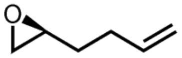

(S)-2-(but-3-en-1-yl)oxirane
(1a)

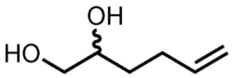

Exact Mass: 116.08
Diol byproduct
(8-rac)

Instrument Type: Agilent 6890 gas chromatograph (GC) with a flame ionization detector (FID)

Conditions:

Column: RT-GammaDEXsa (30 m x 0.25 mm x 0.25 μm)

Inlet Pressure: 16.4 psi | Split Ratio: 50 : 1 | Split Flow: 71.3 mL/min

Column flow: 1.4 mL/min | Inlet Temp: 250°C | Injection volume: 1 μL

Runtime: 16 min

Temperature Program: Isothermal at 60 °C

Sample preparation:

FID Temperature: 250 °C

FID Gas Flows: H_2 = 35.0 mL/min; Air = 450 mL; Makeup (N_2) = 30.0 mL/min

Retention Times

Compound	Time (min)
**(R) enantiomer** – (R)-2-but-3-en-1-yl)oxirane	12.48
**(S) enantiomer** – (S)-2-but-3-en-1-yl)oxirane	13.19

Notes: For accurate integration, draw through entire tail of the (R) peak and tangent skim the (S) peak

Representative Chromatogram(s): *Prepared mixture of 0.5% (S) enantiomer + 99.5% (R) enantiomer*

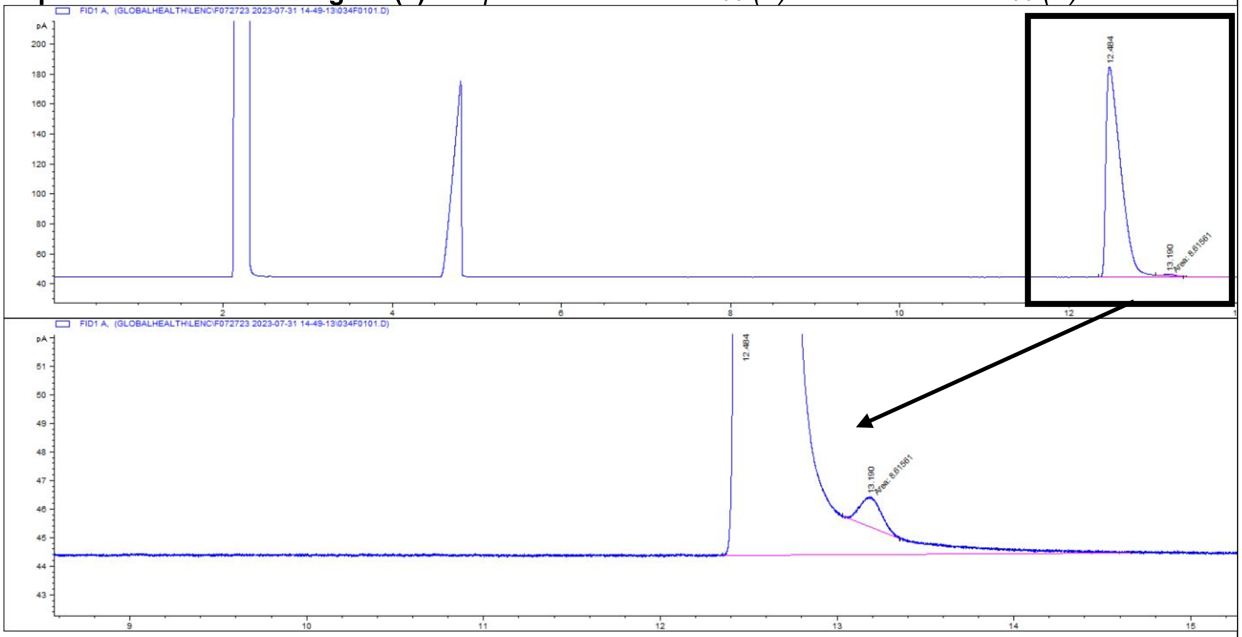

Table S1. Initial optimization of synthesis of ***1-rac*** through epoxidation of 1,5-hexadiene **5**[a]

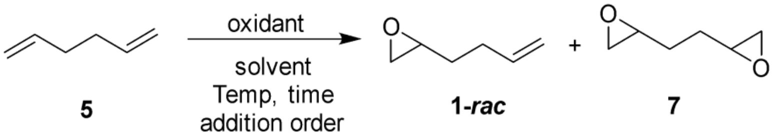

Entry	Oxidant	Solvent (volume)	Addition order[b]	Temp, time	GC-MS analysis (TIC A%)		
					**5**	**1-*rac***	**7**
1	mCPBA	$CHCl_3$ (35V)	Reverse	0 – 19°C, 3h	--	41[c]	--
2	mCPBA	$CHCl_3$ (25V)	Regular	0 – 19°C, 3h	24.4	59.5	16.1
3	mCPBA	DCM (25V)	Regular	0 – 19°C, 3h	20.6	56.9	22.5
4	mCPBA	n-hexane (25V)	Regular	0 – 19°C, 3h	68.4	29.2 (60)[d]	2.4
5	mCPBA	MTBE (25V)	Regular	0 – 19°C, 3h	58.7	36.0 (60)[d]	5.3
6	mCPBA	EtOAc (25V)	Regular	0 – 19°C, 3h	66.2	30.7 (50)[d]	1.7
7	mCPBA	EtOH (25V)	Regular	0 – 19°C, 3h	77.3	22.2 (50)[d]	0.0
8	mCPBA	DCM (25V)	Regular	0 – 2.8°C, 3h	27.6	55.7	16.7
9	^{t}BuOOH	DCM (25V)	Regular	0 –19°C, 24h	100	0	0
10	^{t}BuOOH	n-hexane (25V)	Regular	0 –19°C, 24h	100	0	0

[a]All reactions were performed with 1.0 equivalent of mCPBA and 1.0 equivalent of 1,5-hexadiene. Solvent volume (V) = mL/g of 1,5-hexadiene. All these reactions were monitored by GCMS and reported as TIC A%. [b]Reverse addition: adding 1,5-hexadiene to a solution of mCPBA at 0°C; regular addition: 1,5-hexadiene in 5V of solvent was cooled to 0°C, to this solution was added a solution of mCPBA in 20V of the solvent. [c]Isolated yield after distillation. [d] the TIC A% in parenthesis was obtained by running the reaction for 24h.

DESIGN OF EXPERIMENTS

Design of experiments (DOE) is a valuable tool for designing the systematic table of experiments to investigate the influence of different effective factors including mCPBA/Hexadiene ratio, DCM (volume), Time (min), and Temperature (°C) on the epoxidation of 1,5-hexadiene. Three responses including GCMS TIC area percentage of chemical (**5**), (**1-*rac***), and (**7**) were monitored to find the best-optimized condition of the experiment. A central composite design (CCD) from response surface methodology was used to design the experiments. CCD initializes with two levels, high and low, and then extends the levels of the factors to five by adding +⍰ , -⍰, and center point levels. In general, a CCD for f factors, coded as (x_1, . . . , x_f), consists of three parts. The first part is a factorial (or cubic) design, containing a total of $N_{fact} = 2^f$ points with coordinates $x_i = -1$ or $x_i = +1$, for i=1, ..., f. Second is an axial (or star) part formed by $N_{ax} = 2 \times f$ points with all their coordinates null except for the one that is set equal to a certain value +⍰ (or −⍰), which usually ranges from 1 to $\sqrt{f}$. Third is a total of N_c runs performed at the center point of the experimental region, where x_1=x_2= ... =x_f=0. In this study, the number of center points was 3, and a rotatable CCD with ⍰=1.68 was used to design the experiments, which resulted in 27 experiments. Table S2 shows the details about CCD factors, and their levels, and Table S3 shows the designed table of experiments. Experiment 4, in Table S3, was considered as one of the optimized conditions for epoxidation of 1,5-hexadiene with having 92% corrected assay yield based on GC-MS. In this regard, without any further multivariate optimization within the DOE experiments, one of the optimized conditions for epoxidation of 1,5-hexadiene was discovered.

Table S2. Details about CCD factors for epoxidation of 1,5-hexadiene (**5**)

**Factor (Units)**	**Minimum**	**Maximum**	**Coded Low**	**Coded High**	**Mean**
Factor A: mCPBA/hexadiene	0.4	2.0	-1 ↔ 0.80	+1 ↔ 1.60	1.2
Factor B: DCM (mL)	5	25	-1 ↔ 10	+1 ↔ 20	15
Factor C: Time (min)	20	180	-1 ↔ 60	+1 ↔ 140	100
Factor D: Temperature (°C)	-11	29	-1 ↔ -1	+1 ↔ 19	9

Table S3. Designed table of experiments for epoxidation of 1,5-hexadiene (**5**)[a]

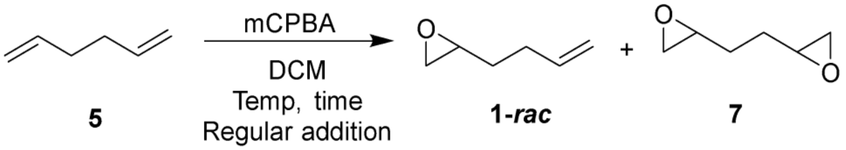

Entry	mCPBA /Hexadiene ratio	DCM (volume)	Time (min)	Temp (°C)	GC-MS analysis (TIC A%)			Corrected assay yield based on GC-MS
					(5)	(1-*rac*)	(7)	
1	0.8	20	140	-1	45.70	46.30	8.00	57%
2	1.6	20	140	-1	38.13	50.19	11.68	50%
3	1.6	20	140	19	2.46	39.68	57.86	39%
4	0.4	15	100	9	58.42	37.39	4.20	92%
5	1.2	5	100	9	16.27	54.82	28.90	54%
6	2	15	100	9	5.95	48.18	45.86	48%
7	1.6	10	140	19	1.78	37.23	60.99	37%
8	0.8	10	60	-1	62.94	34.46	2.60	42%
9	1.6	20	60	19	7.79	50.31	41.91	50%
10	0.8	10	140	19	30.88	52.22	16.90	65%
11	1.2	15	100	9	20.21	54.64	25.15	54%
12	0.8	20	140	19	30.58	52.16	17.46	65%
13	1.2	15	20	9	46.39	45.73	7.87	45%
14	1.2	15	100	-11	73.91	24.99	1.10	25%
15	0.8	20	60	-1	64.89	32.82	2.29	40%
16	0.8	10	60	19	29.75	53.29	16.95	66%
17	1.2	15	100	9	20.23	54.94	24.83	55%

18	1.2	15	100	29	12.95	53.33	33.72	53%
19	1.6	10	60	19	4.39	45.67	49.95	45%
20	0.8	20	60	19	34.74	51.58	13.68	63%
21	0.8	10	140	-1	45.01	42.57	12.42	52%
22	1.6	10	140	-1	44.53	46.42	9.05	46%
23	1.6	10	60	-1	64.91	32.67	2.41	32%
24	1.2	15	100	9	20.41	54.90	24.69	55%
25	1.6	20	60	-1	29.81	63.52	6.68	63%
26	1.2	25	100	9	22.58	54.53	22.89	54%
27	1.2	15	180	9	15.24	54.38	30.38	54%

[a]All reactions were performed with 1g of 1,5-dihexadiene, monitored by GCMS and reported as TIC A%. Solvent volume (V) = mL/g of 1,5-hexadiene. regular addition: 1,5-hexadiene in 5V of solvent was cooled to 0°C, to this solution was added solid mCPBA in one portion.

Further multivariate optimization has resulted in the six interaction terms between every two factors of A-B, A-C, A-D, B-C, B-D, and C-D that affect R2 (**1-*rac***) are shown below (Figure S1).

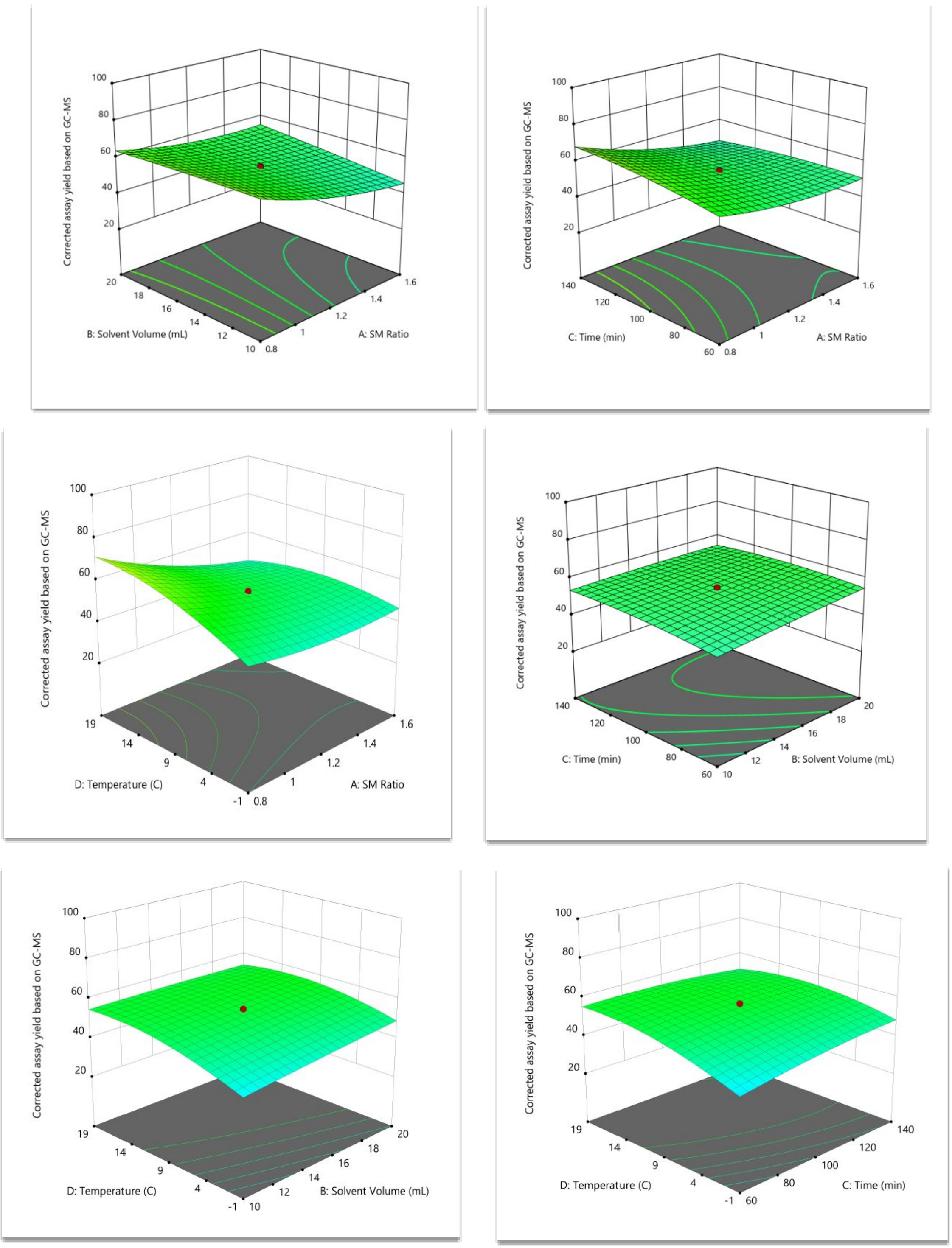

Figure S1. 3-D plot of the primary interactions (A-B, A-C, A-D, B-C, B-D, C-D).

According to the ANOVA table:

- A, D, AD, and D² are significant model terms by having p-values less than 0.05 (95% confidence level) which means they are the influential factors (please see the red boxes).
- CD and A² have p-values very close to 0.05, so they also could be considered extra-influential factors (please see the purple boxes).
- The model is significant because its p-value is less than 0.05 which is very good and its p-value is very low (0.0017). The lower the p-value, the more significant and better (please see the green box).
- The Lack of fit is significant because its p-value is less than 0.05 which is bad for the model and it means there is a systematic error with the model, but this is not true because it is the software's bug and drawback (please see the yellow box). The Lack of fit should not be significant and its p-value should be greater than 0.05. The value of the p-value for the Lack of fit is the opposite of the factors and model. The software's bugs and drawbacks come from the three repeated center points. In the ideal case, the response of the three repeated center points should be the same or very close which shows repeatability but it is surprising in the software they should be a little different from each other. The experiments 11, 17, and 24 are the three center points (all factors are the same) and their corrected assay yield based on GC-MS are 54%, 55% and 55% respectively, to fix this software bug, I changed their corrected assay yield based on GC-MS to 53%, 55%, and 57% respectively, and after that I repeated the optimization. In this way, the Lack of fit is not significant because its p-value is more than 0.05. This is the proof of that, there is no systematic error in the experiments and its software bug. I put the new fake ANOVA table (yellow box) beside the true one (purple box).

ANOVA for Quadratic model

Response 4: Corrected assay yield based on GC-MS
Transform: Square Root
Constant: 0

Source	Sum of Squares	df	Mean Square	F-value	p-value	
Model	18.26	14	1.30	6.11	0.0017	significant
A-SM Ratio	5.53	1	5.53	25.91	0.0003	
B-Solvent Volume	0.3978	1	0.3978	1.86	0.1974	
C-Time	0.1850	1	0.1850	0.8661	0.3704	
D-Temprature	2.56	1	2.56	11.99	0.0047	
AB	0.6000	1	0.6000	2.81	0.1196	
AC	0.6444	1	0.6444	3.02	0.1080	
AD	2.22	1	2.22	10.39	0.0073	
BC	0.1426	1	0.1426	0.6677	0.4298	
BD	0.3640	1	0.3640	1.70	0.2162	
CD	0.8114	1	0.8114	3.80	0.0750	
A²	0.8348	1	0.8348	3.91	0.0715	
B²	0.0193	1	0.0193	0.0902	0.7691	
C²	0.2585	1	0.2585	1.21	0.2929	
D²	2.35	1	2.35	11.02	0.0061	
Residual	2.56	12	0.2136			
Lack of Fit	2.56	10	0.2560	167.43	0.0060	significant
Pure Error	0.0031	2	0.0015			
Cor Total	20.82	26				

ANOVA for Quadratic model

Response 4: Corrected assay yield based on GC-MS
Transform: Square Root
Constant: 0

Source	Sum of Squares	df	Mean Square	F-value	p-value	
Model	18.29	14	1.31	6.04	0.0017	significant
A-SM Ratio	5.53	1	5.53	25.57	0.0003	
B-Solvent Volume	0.3978	1	0.3978	1.84	0.2001	
C-Time	0.1850	1	0.1850	0.8550	0.3734	
D-Temprature	2.56	1	2.56	11.83	0.0049	
AB	0.6000	1	0.6000	2.77	0.1217	
AC	0.6444	1	0.6444	2.98	0.1100	
AD	2.22	1	2.22	10.26	0.0076	
BC	0.1426	1	0.1426	0.6591	0.4327	
BD	0.3640	1	0.3640	1.68	0.2190	
CD	0.8114	1	0.8114	3.75	0.0767	
A²	0.7896	1	0.7896	3.65	0.0803	
B²	0.0269	1	0.0269	0.1241	0.7307	
C²	0.2847	1	0.2847	1.32	0.2737	
D²	2.43	1	2.43	11.24	0.0058	
Residual	2.60	12	0.2164			
Lack of Fit	2.56	10	0.2560	14.08	0.0681	not significant
Pure Error	0.0364	2	0.0182			
Cor Total	20.89	26				

R²	0.8769
Adjusted R²	0.7333
Predicted R²	0.2914
Adeq Precision	10.8381

R²	0.8757
Adjusted R²	0.7307
Predicted R²	0.2901
Adeq Precision	10.7683

- As it can be seen in the both ANOVA tables the p-values of other terms and the model are very similar and just the problem of the lack of fit is fixed by changing the three center points.
- In both cases instead of the corrected assay yield based on GC-MS, the square root of the corrected assay yield based on GC-MS is modeled and Quadratic model was used.
- As you can see the blow of the ANOVA table I put the fitting statistics. In both cases, the value of R^2, Adjusted R^2, and Predicted R^2 are similar.
- The value of R^2 is saying that around 87% of the variance in the dependent variable that is predictable from the independent variables. However, the value of the Adjusted R^2 (73%) implies there should be an outlier in the experiments because of that the R^2 and Adjusted R^2 are different. The value of the Adjusted R^2 and Predicted R^2 are very more than expected different which implies there is a blocking effect in the experiments.
- The Adeq Precision is representative of the signal to noise ration and its value around 10 is good enough to be reliable.
- The equation in terms of coded factors can be used to make predictions about the response for given levels of each factor and with the coded equation, we can identify the relative impact of the factors by comparing the factor coefficients.

Final Equation in Terms of Coded Factors

Sqrt(Corrected assay yield based on GC-MS) =

+7.39	
-0.4802	A
+0.1287	B
+0.0878	C
+0.3266	D
+0.1937	AB
-0.2007	AC
-0.3725	AD
-0.0944	BC
-0.1508	BD
-0.2252	CD
+0.1978	A^2
-0.0300	B^2
-0.1101	C^2
-0.3321	D^2

Final Equation in Terms of Coded Factors

Sqrt(Corrected assay yield based on GC-MS) =

+7.42	
-0.4802	A
+0.1287	B
+0.0878	C
+0.3266	D
+0.1937	AB
-0.2007	AC
-0.3725	AD
-0.0944	BC
-0.1508	BD
-0.2252	CD
+0.1924	A^2
-0.0355	B^2
-0.1155	C^2
-0.3376	D^2

- As it can be seen in the coded version of the equations, the coefficients of the equation terms are very similar as well.
- For seek of the factor importance, based on the p-values, A, D, AD, and D^2 were significant factors in the model and their corresponding coefficients are around -0.48, +0.33, -0.37, and -0.33. The negative and positive sign of each factor implies their negative and positive contributions respectively on the overall value of the equation.
- The most influential factor is Factor "A" with the absolute value of 0.48, and then Factor "AD" with the absolute value of 0.37 and Factors "D" and "D^2" has the same absolute value of 0.33.
- The relative importance of the four main factors (A, B, C, D), the six interaction factors (AB, AC, AD, BC, BD, CD), the four power 2 of factors (A^2, B^2, C^2, D^2) can be find and sort by using their coded coefficients in the above equation. For example, for the four main factors, the relative importance is A>D>B>C.

- It should be noted that there is probably an outlier in the system that caused the Adjusted R^2 to be different from R^2. Some statistical tests showed that experiment 25 is one of the most probable outliers which has a red circle. Experiments 10, and 17 also are two less probable ones.

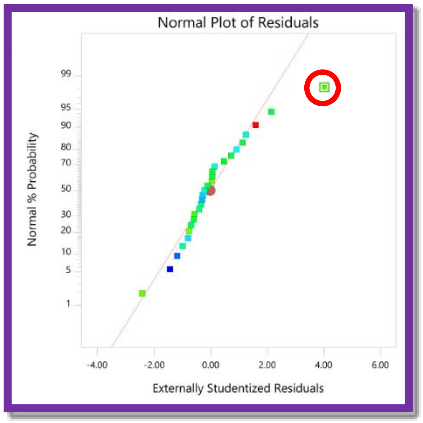

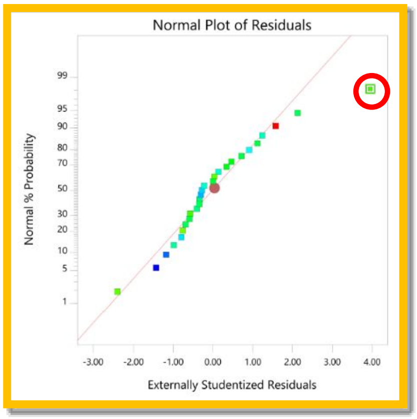

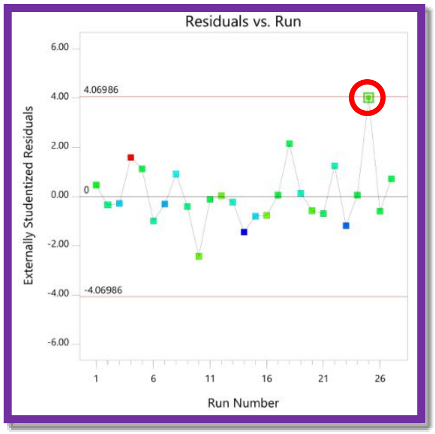

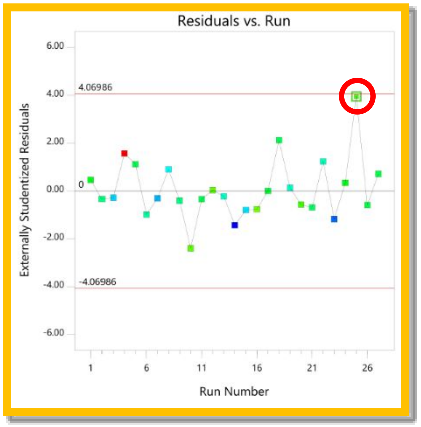

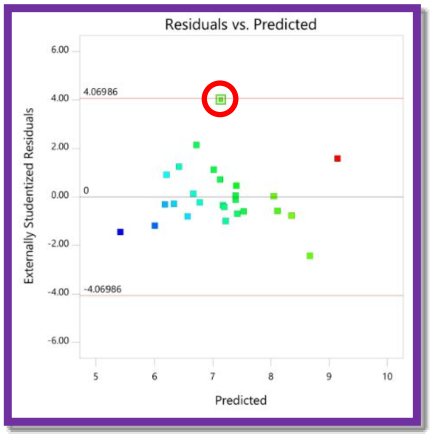

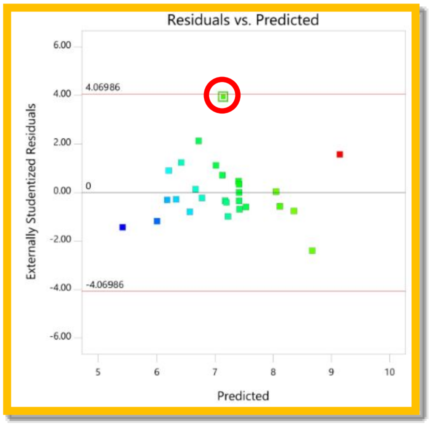

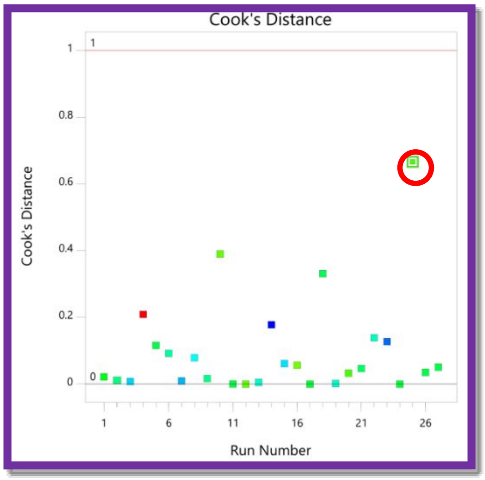

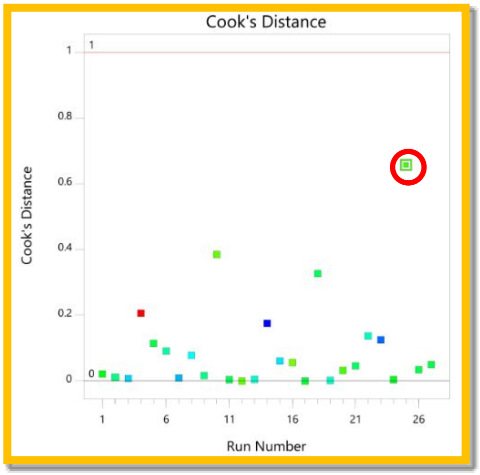

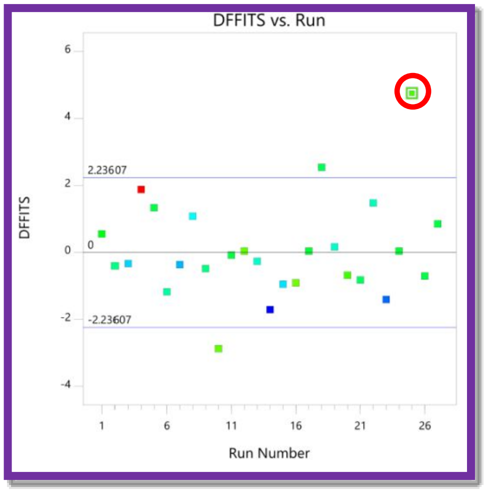

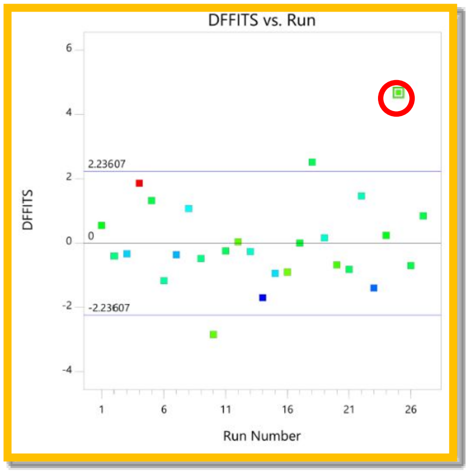

- In following the perturbation, one factor, and two-factor interactions figures are presented.

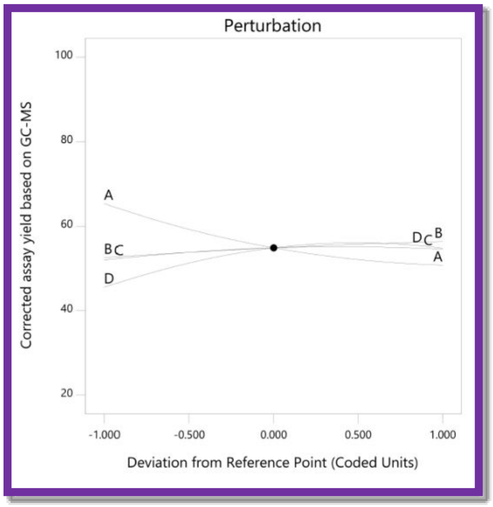

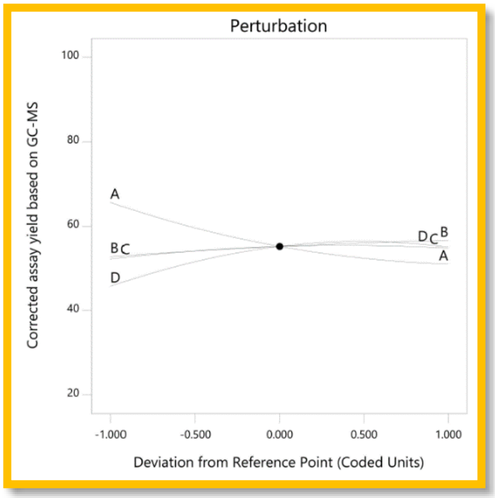

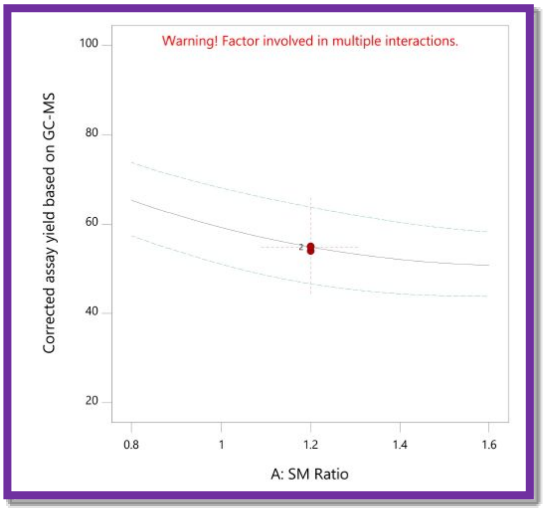
Warning! Factor involved in multiple interactions.
Corrected assay yield based on GC-MS
A: SM Ratio

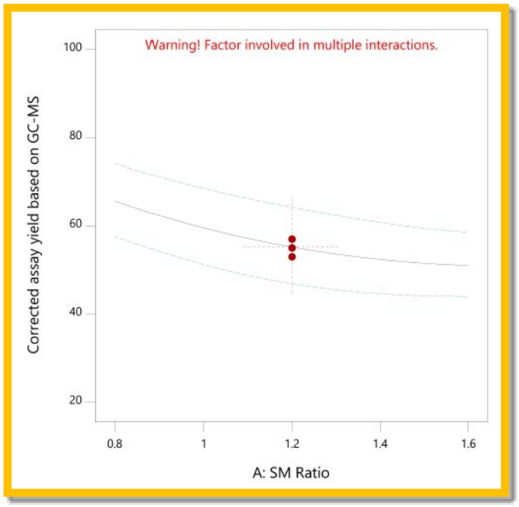
Warning! Factor involved in multiple interactions.
Corrected assay yield based on GC-MS
A: SM Ratio

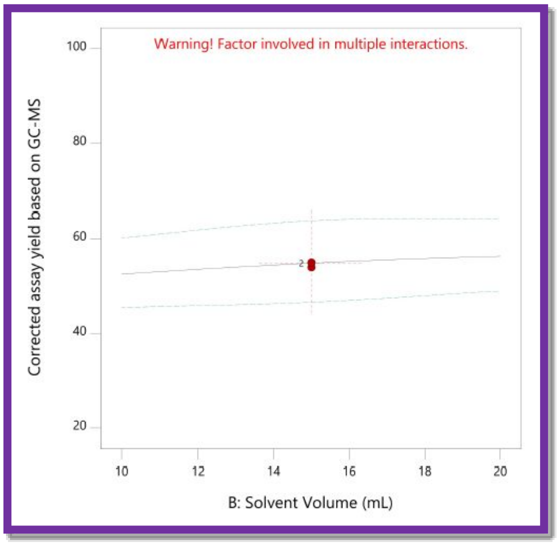
Warning! Factor involved in multiple interactions.
Corrected assay yield based on GC-MS
B: Solvent Volume (mL)

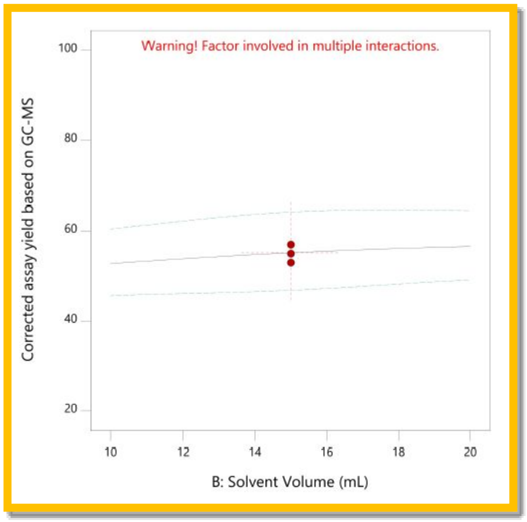
Warning! Factor involved in multiple interactions.
Corrected assay yield based on GC-MS
B: Solvent Volume (mL)

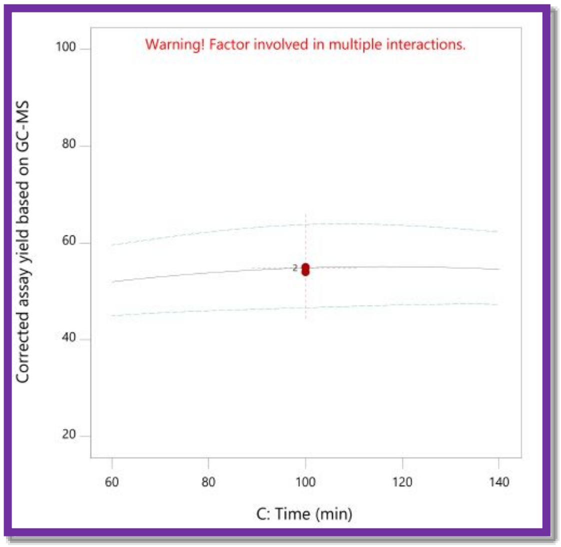
Warning! Factor involved in multiple interactions.
Corrected assay yield based on GC-MS
C: Time (min)

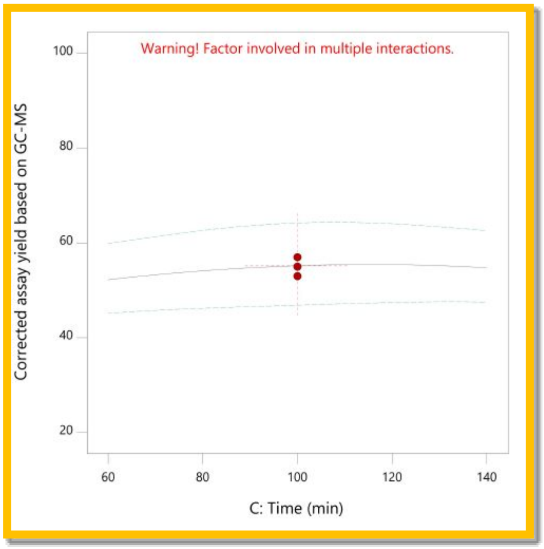
Warning! Factor involved in multiple interactions.
Corrected assay yield based on GC-MS
C: Time (min)

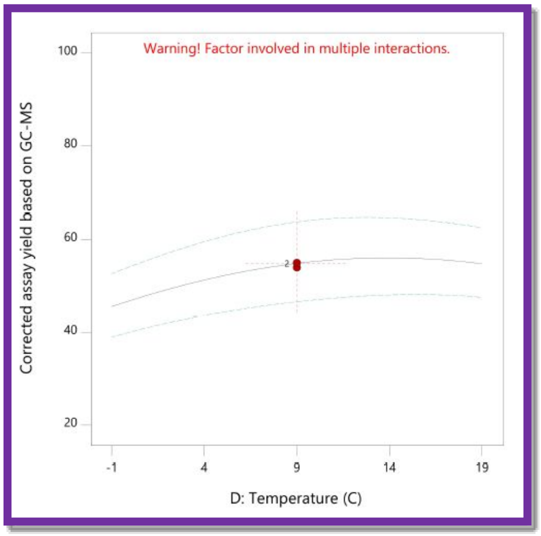
Warning! Factor involved in multiple interactions.
Corrected assay yield based on GC-MS
D: Temperature (C)

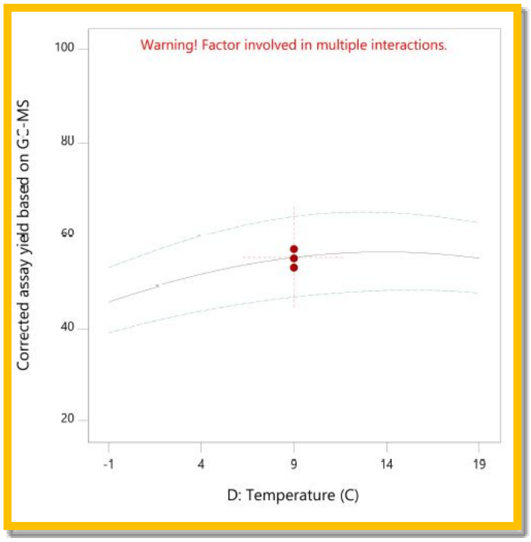
Warning! Factor involved in multiple interactions.
Corrected assay yield based on GC-MS
D: Temperature (C)

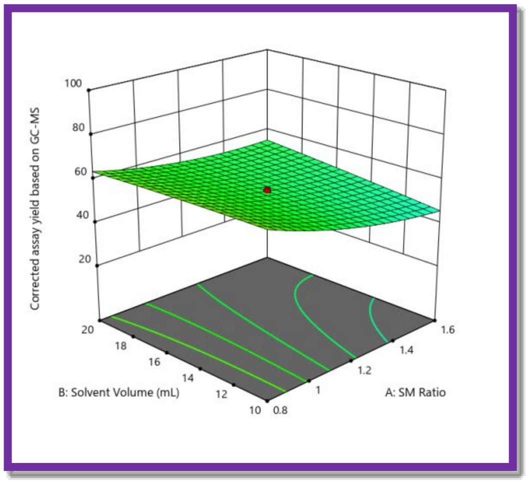
Corrected assay yield based on GC-MS
B: Solvent Volume (mL)
A: SM Ratio

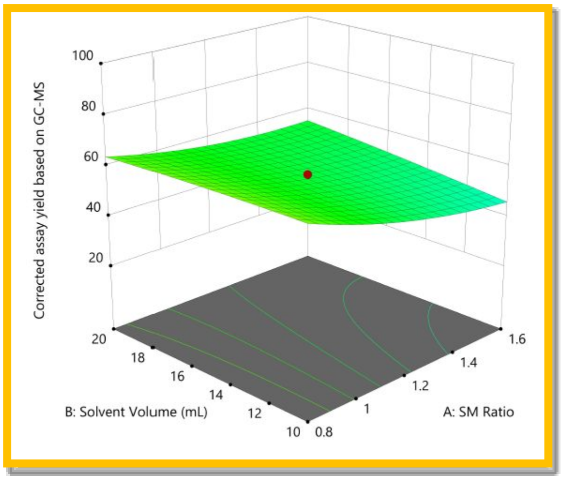
Corrected assay yield based on GC-MS
B: Solvent Volume (mL)
A: SM Ratio

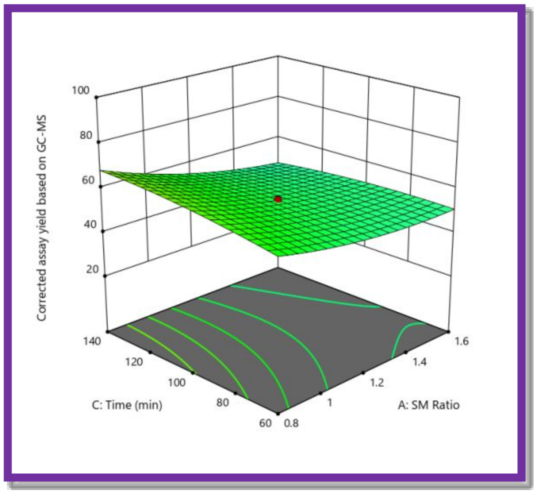
Corrected assay yield based on GC-MS
C: Time (min)
A: SM Ratio

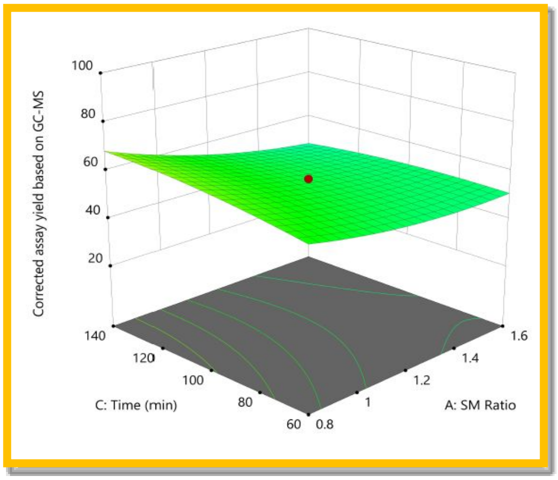
Corrected assay yield based on GC-MS
C: Time (min)
A: SM Ratio

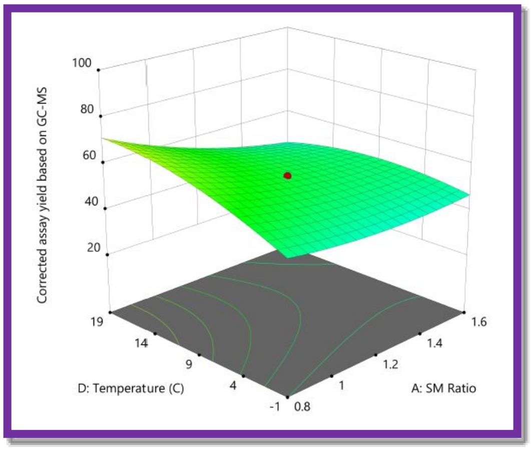

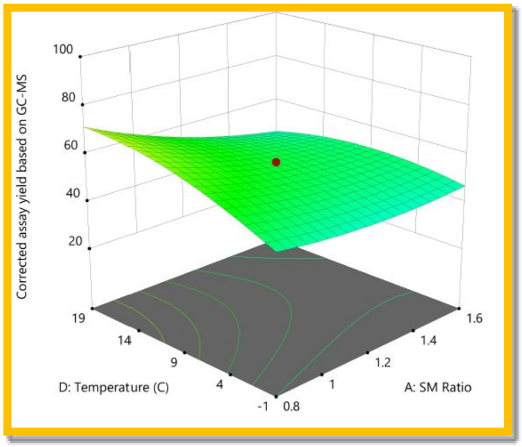

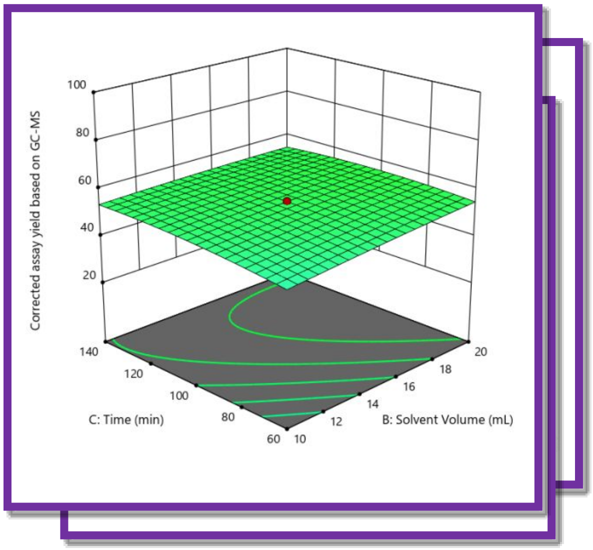

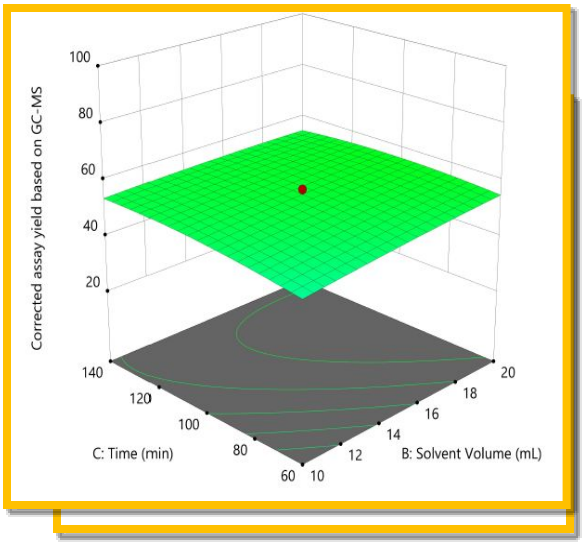

- ➢ In the below figures, the predicted versus actual values are shown.

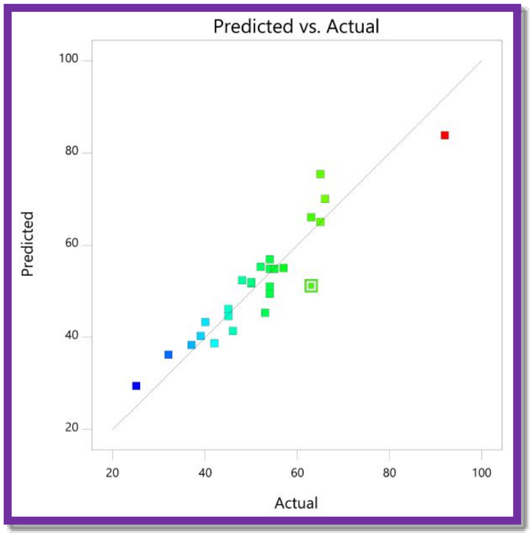

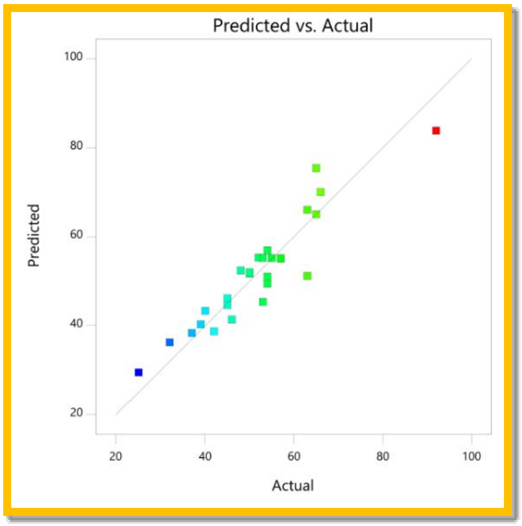

- According to the aforementioned results, it concludes that there was no difference between the results and figures when changing the two out of three center point values, which indicated that these results are data understanding and not data manipulation.

Table S4. Further optimization of epoxidation of 1,5-hexadiene (**5**) for synthesis of epoxide **1-*rac***[a]

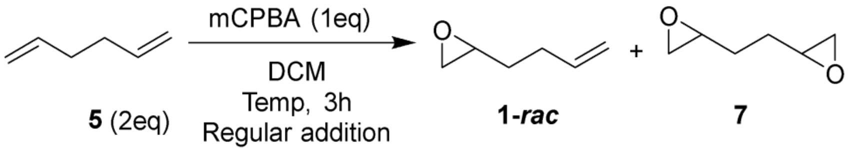

Entry	DCM (volume)	Temp (°C)	GC-MS analysis (TIC A%)			Corrected assay yield
			**5**	**1-*rac***	**7**	
1	2	0	67.0	30.4	2.6	60.8%
2	5		57.7	37.6	4.7	75.2%
3	10		57.2	37.8	5.0	75.6%

4	15		56.0	38.4	5.6	76.8%
5	2		53.6	38.8	7.6	77.6%
6	5	10	53.0	40.0	7.0	80.0%
7	10		51.4	41.6	7.0	83.2%
8	15		51.9	41.4	6.8	82.8%
9[b]	5	0	50.2	42.9	6.8	85.8%

[a]All reactions were performed with 1.0g of 1,5-hexadiene (2.0 equivalents) and 1.0 equivalent of mCPBA with a regular addition at the temperature as indicated in the table for 3h. Solvent volume (V) = mL/g of 1,5-hexadiene. The reaction was maintained at the desired temperature using an immersion chiller. All the reactions were monitored by GCMS and reported as TIC A%. [b]Run at 25g scale, 50% isolated yield after distillation together with >90% of recovery of 1,5-hexadiene.

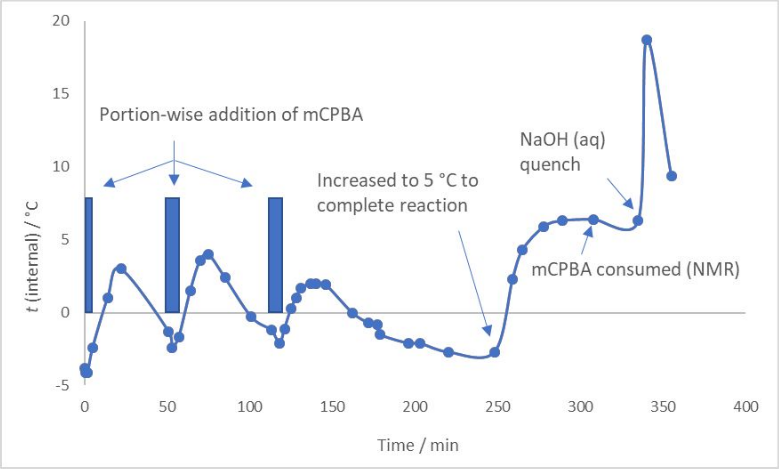

Figure S2. Plot of the epoxidation of 1,5-hexadiene (**5**) performed at 150g scale of 1,5-hexadiene with 0.5 equivalent of mCPBA (210g) in three equal portions with 5V of DCM (750 mL) as a solvent. Solvent volume (V) = mL/g of 1,5-hexadiene. See Experimental Procedure section and Table 1, entry 2 in the manuscript for more details.

It is worth mentioning that the control of the internal temperature of the epoxidation below 5°C was critical to minimize the formation of the diepoxide in scale. The internal temperature of the epoxidation was closely monitored as exemplified in one of the 150g scale reactions (Figure S2). The jacket temperature was setup at -10°C. The internal temperature of the reaction mixture was below 5°C if adding 70g of mCPBA in one portion with a 50min interval. Another internal temperature spike was observed during the NaOH (2.5N) quench. The temperature was increased to 20°C during the neutralization. Notably, any unconsumed mCPBA may cause a safety concern at scale, especially when distillation was used for purification. Hence, we monitored mCPBA

consumption by crude ^{1}H NMR as well as a peroxide strip test. Under the condition, the utilization of 2.0 equivalents of 1,5-hexadiene ensured a complete consumption of mCPBA within 3h. Additionally, the following quench with NaOH (2.5N) was further convinced the consumption of any remaining mCPBA. It was visualized with a clear organic and aqueous phases formation after treating with NaOH (2.5N), indicating the complete basifying of the carboxylic acid as well as any possible remaining mCPBA. And the separated organic phase was safe for the next distillation.

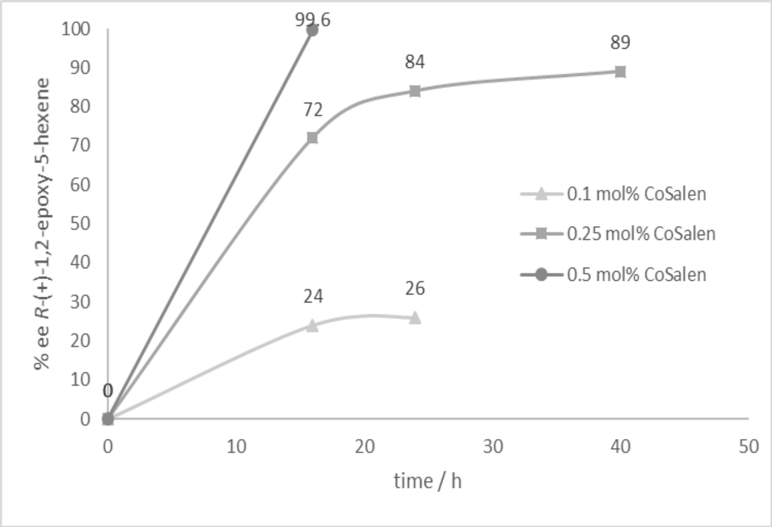

Figure S3. Catalyst loading variations for the hydrolytic kinetic resolution of rac-1,2-epoxy-5-hexene **1-*rac***. Conditions: **1-*rac*** (1g), (*R*,*R*)-(salen)Co(II), AcOH (2mol%), THF (0.1mL), air, 0°C-rt, water (0.55equiv); monitored by chiral GC-FID.

EXPERIMENTAL PROCEDURE

Synthesis of rac-(±)-1,2-epoxy-5-hexene (1-rac) (step **1a**, *resolution route)*

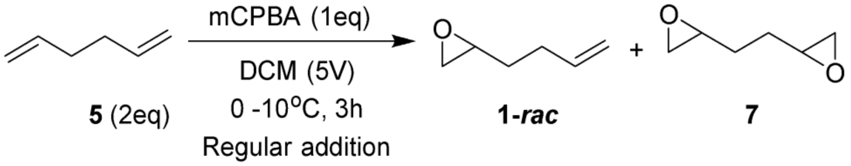

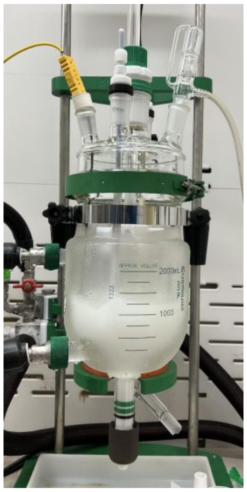
Reaction mix

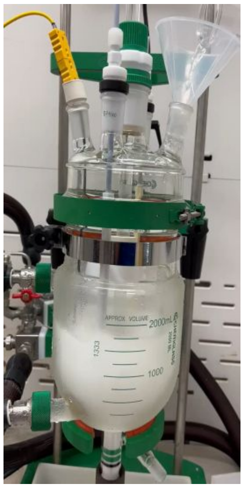
NaOH quench

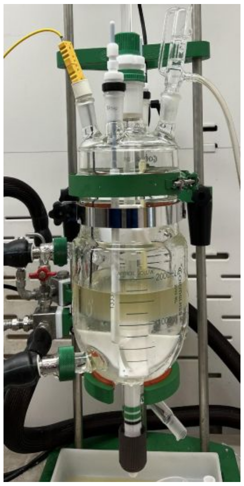
Clear phase separation

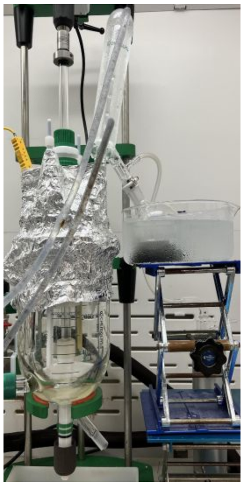
Distillation

A 2L ChemRxnHub reactor was charged with 750 mL DCM (5V) and 1,5-hexadiene (150g, 1.83mol, 2 equiv), and the reaction solution was cooled to -5°C (internal temperature -3.8°C) with a chiller under aerobic conditions. Solid mCPBA (210.0g, 912.7mmol, 1 equiv) was added in three equal portions (3 × 70.0 g), maintaining the internal temperature < 5°C. Once the reaction cooled back down to -3°C after the final addition, the reaction was assayed for unconsumed mCPBA: ca. 10% mCPBA remained (^{1}H NMR, CD_3CN). The reaction was warmed to 5°C and stirred for 1h to complete. At which point the reaction was quenched with aq. NaOH (440mL, 2.5N, 0.6equiv), stirred briefly, separated, and the organic phase was assayed for product epoxide (86.83g, 97%). The epoxide solution was concentrated to ca. 250mL at 65°C, and further distillation of the volatiles continued at 50-80°C under gentle N_2 stream (0.1 NL/min) on a separate distillation setup with a 10” Vigreaux column, long-path condenser into a cooled (-78°C) receiving flask to recover 1,5-hexadiene (71g, yield: 95%) as a solution in DCM. Once the volatiles were purged, atmospheric distillation continued at 170-200°C to yield *rac*-1,2-epoxy-5-hexene **1-*rac*** (67.5g, 93.8wt%, yield: 71%).

^{1}H NMR (600 MHz, $CDCl_3$) δ 5.83 (ddt, *J* = 16.9, 10.2, 6.6 Hz, 1H), 5.02 (dddd, *J* = 10.2, 8.9, 5.3, 3.8 Hz, 1H), 4.98 (ddd, *J* = 10.2, 3.0, 1.3 Hz, 1H), 2.99 – 2.83 (m, 1H), 2.74 (dd, *J* = 4.8, 4.2 Hz, 1H), 2.47 (dd, *J* = 5.0, 2.7 Hz, 1H), 2.32 – 2.12 (m, 2H), 1.68 – 1.35 (m, 2H).

^{13}C NMR (150 MHz, $CDCl_3$) δ 137.6, 115.1, 51.8, 47.1, 31.8, 30.2.

MS-EI (*m/z*) (M^+): 98.1.

Synthesis of R-(+)-1,2-epoxy-5-hexene (1) (step 2a, resolution route)

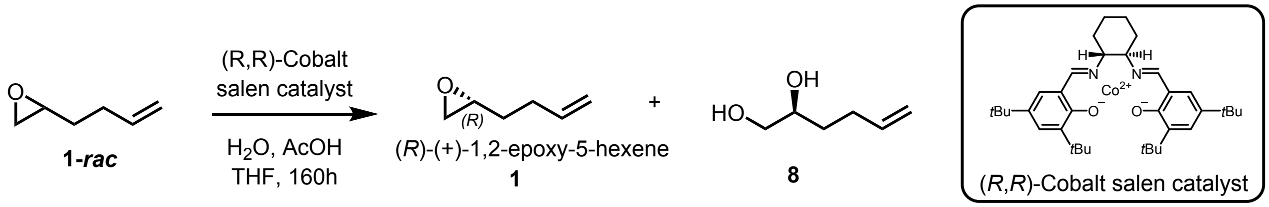

A 250 mL flask with over-head stirring was charged with (*R*,*R*)-(salen)Co(II) (1.82g, 3.01mmol, 0.005equiv). The catalyst was treated with *rac*-1,2-epoxy-5-hexene **1-*rac*** (63.1g, 93.8wt%, 602.8 mmol), AcOH (0.69mL, 12.06mmol, 0.02equiv), and 6mL of THF under aerobic conditions. The reaction flask was cooled to 0˚C, and H_2O (6.0mL, 332mmol, 0.55equiv) was added in one portion. The reaction was allowed to warm to room temperature and monitored by chiral GC-FID. After stirring for 160h, the ee was 94%, with an assay yield of 49%. At this time the volatile materials were distilled at 90˚C under a gentle N_2 stream (0.1 NL/min), followed by vacuum transfer under 90Torr at 90˚C to afford (*R*)-1,2-epoxy-5-hexene **1** (19.82g, 602.8mmol, 33.5%).

^{1}H NMR (600 MHz, $CDCl_3$) δ 5.79 (ddt, *J* = 16.9, 10.2, 6.6 Hz, 1H), 5.00 (dd, *J* = 17.1, 1.7 Hz, 1H), 4.97 – 4.88 (m, 1H), 3.07 – 2.74 (m, 1H), 2.69 (dd, *J* = 4.8, 4.3 Hz, 1H), 2.42 (dd, *J* = 5.1, 2.7 Hz, 1H), 2.28 – 2.03 (m, 2H), 1.69 – 1.35 (m, 2H).

^{13}C NMR (150 MHz, $CDCl_3$) δ 137.6, 115.1, 51.8, 47.1, 31.8, 30.1.

MS-EI (*m/z*) (M^+): 98.1.

Synthesis of (R)-1-chlorohex-5-en-2-ol (10) (step 1b, epichlorohydrin route)

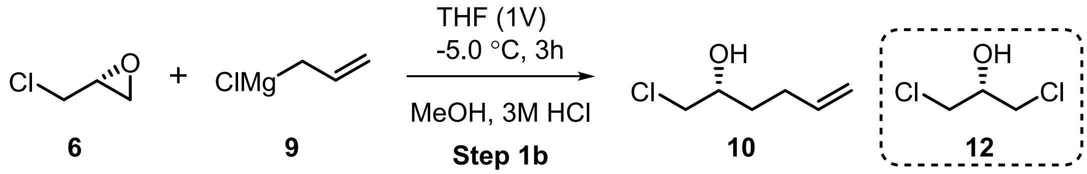

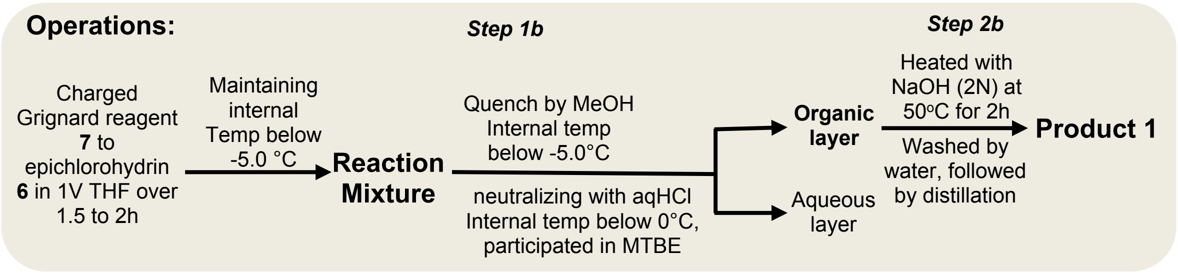

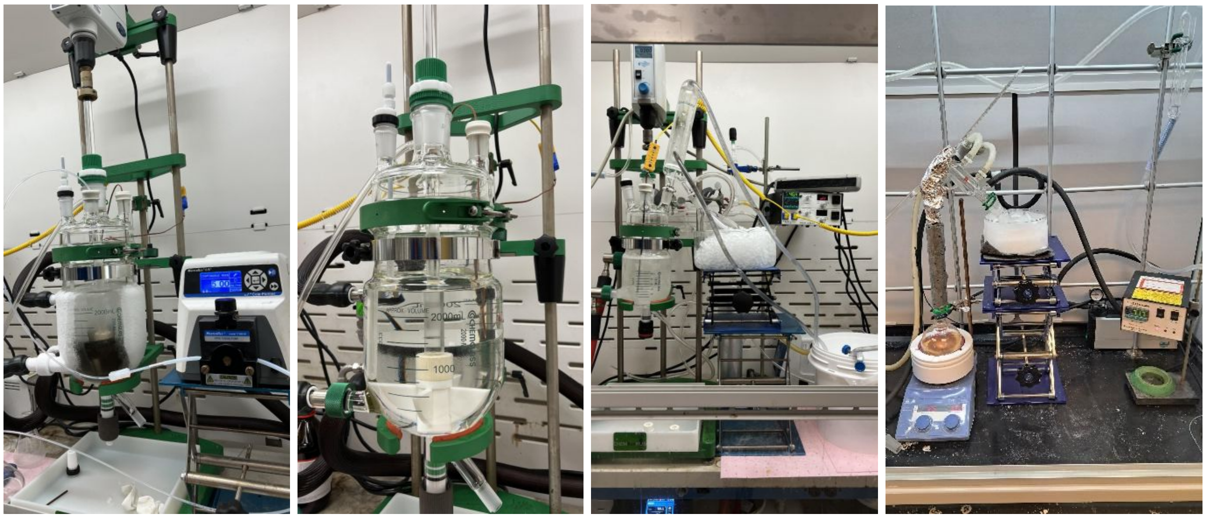

Reaction Clear phase separation MTBE Removal Distillation

To a 5L ChemRxnHub reactor under a nitrogen atmosphere, THF (200mL, 1V) was added followed by (*R*)-epichlorohydrin **6** (200g, 2.16mol, 1eq). This mixture was cooled at -25˚C (internal temperature was -15.5˚C) using a chiller. When the internal temperature achieved -15˚C, allylmagnesium chloride **9** (1.08L, 2.16mol, 1eq, 2M in THF) was added using a peristaltic pump with a flow rate of 5-10 mL/min, maintaining the internal temperature below -5.0˚C. After addition, this mixture was stirred at the same temperature for an additional 1h. Then methanol (219mL, 5.4mol, 2.5eq) was added dropwise, keeping the internal temperature below 0˚C, followed by addition of HCl (2.16L, 2M, 2.0eq) at 0˚C. After that, the circulating cooling system

was turned off and MTBE (1L) was added. The organic layer was collected and washed with HCl (400mL, 2M) and water (400mL), respectively. This resulting organic layer (1.8L) gave an in-solution yield of 91% **10** assayed by GCMS, containing 4% of dichlorohydrin **12** and 0.4% of 1,8-nonadien-5-ol **11**. The crude of compound **10** was used for the next step without further purification.

^{1}**H NMR** (600 MHz, $CDCl_3$) δ 5.81 (ddt, *J* = 16.9, 10.2, 6.7 Hz, 1H), 5.05 (dd, *J* = 17.1, 1.3 Hz, 1H), 4.99 (d, *J* = 10.2 Hz, 1H), 3.94 – 3.75 (m, 1H), 3.62 (dd, *J* = 11.1, 3.4 Hz, 1H), 3.48 (dd, *J* = 11.1, 7.0 Hz, 1H), 2.33 (d, *J* = 3.8 Hz, 1H), 2.25 – 2.08 (m, 2H), 1.71 – 1.54 (m, 2H).

^{13}**C NMR** (150 MHz, $CDCl_3$) δ 137.8, 115.5, 70.9, 50.5, 33.4, 29.8.

MS-EI (*m/z*) (M^+): 134.1.

Synthesis of R-(+)-1,2-epoxy-5-hexene (1) (step 2b, epichlorohydrin route)

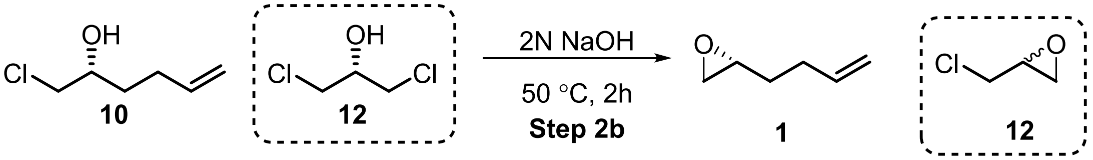

A 5L ChemRxnHub reactor was charged with a solution of chlorohydrin **10** in MTBE (1.8L). To the reactor was added an aqueous solution of NaOH (1.3L, 1.2eq, 2N). The mixture was heated to 50°C and stirred for 2h. After completion, the organic layer was collected and washed with water (500mL × 4) until the aqueous measured pH = 7. The resulting organic phase gave an in-solution yield of 77% assayed by GCMS, containing chiral epoxide **1** of 98.6%A and epichlorohydrin **6-*rac*** of 1.5%A. The solution was evaporated at 90°C to remove solvents of MTBE and THF. The resulting crude product was distilled at 130-170°C to afford the desired epoxide **1** (125g, yield: 59%, purity: 99%A by GCMS, ee: 99.9%).

1H NMR (600 MHz, $CDCl_3$) δ 5.82 (ddt, *J* = 13.1, 10.0, 6.6 Hz, 1H), 5.04 (d, *J* = 17.1 Hz, 1H), 4.97 (d, *J* = 10.2 Hz, 1H), 2.98 – 2.88 (m, 1H), 2.73 (t, *J* = 4.4 Hz, 1H), 2.54 – 2.42 (m, 1H), 2.30 – 2.12 (m, 2H), 1.70 – 1.52 (m, 2H).

13C NMR (150 MHz, $CDCl_3$) δ 137.7, 115.2, 51.9, 47.2, 31.9, 30.2.

MS-EI (*m/z*) (M^+): 98.1.

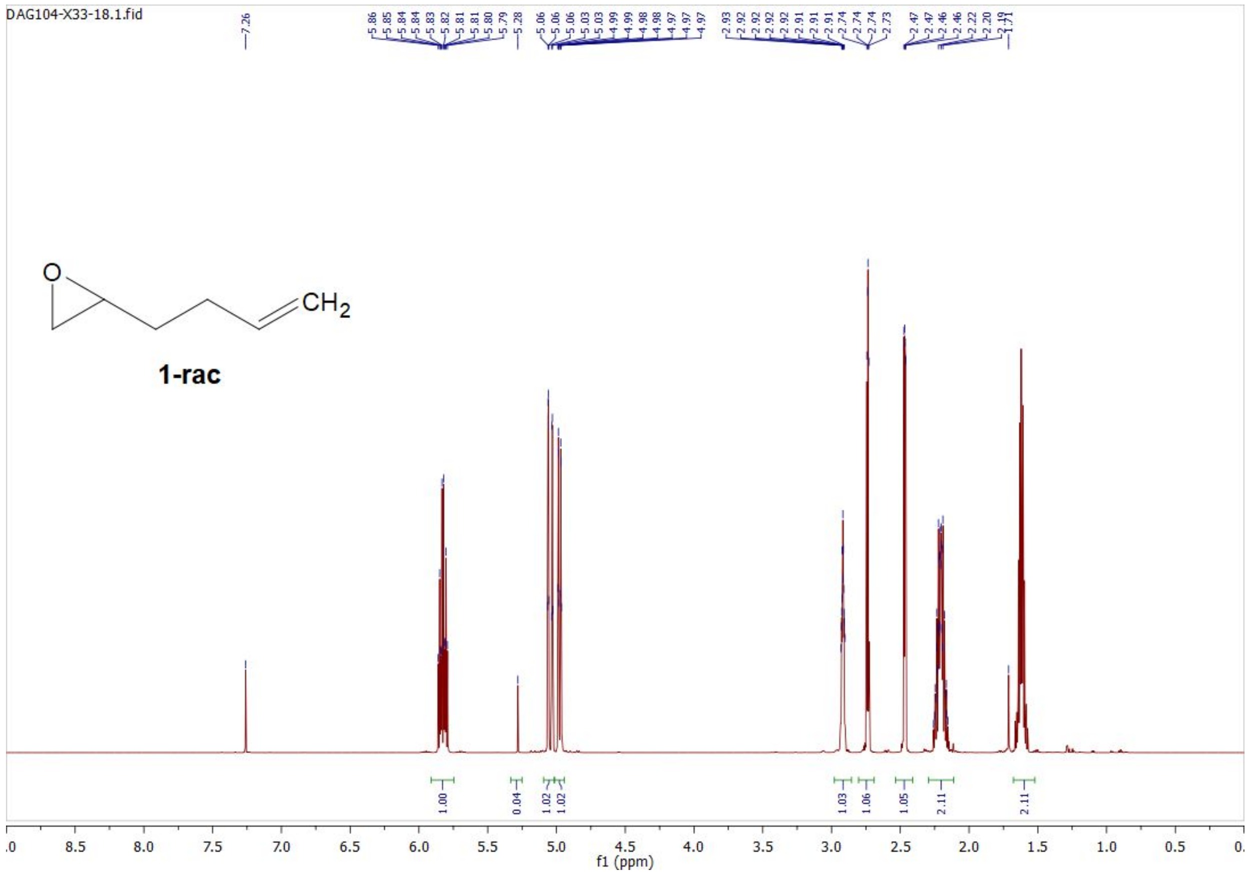

Figure S4. ^{1}HNMR (600MHz, $CDCl_3$) of **1-rac**

10 1

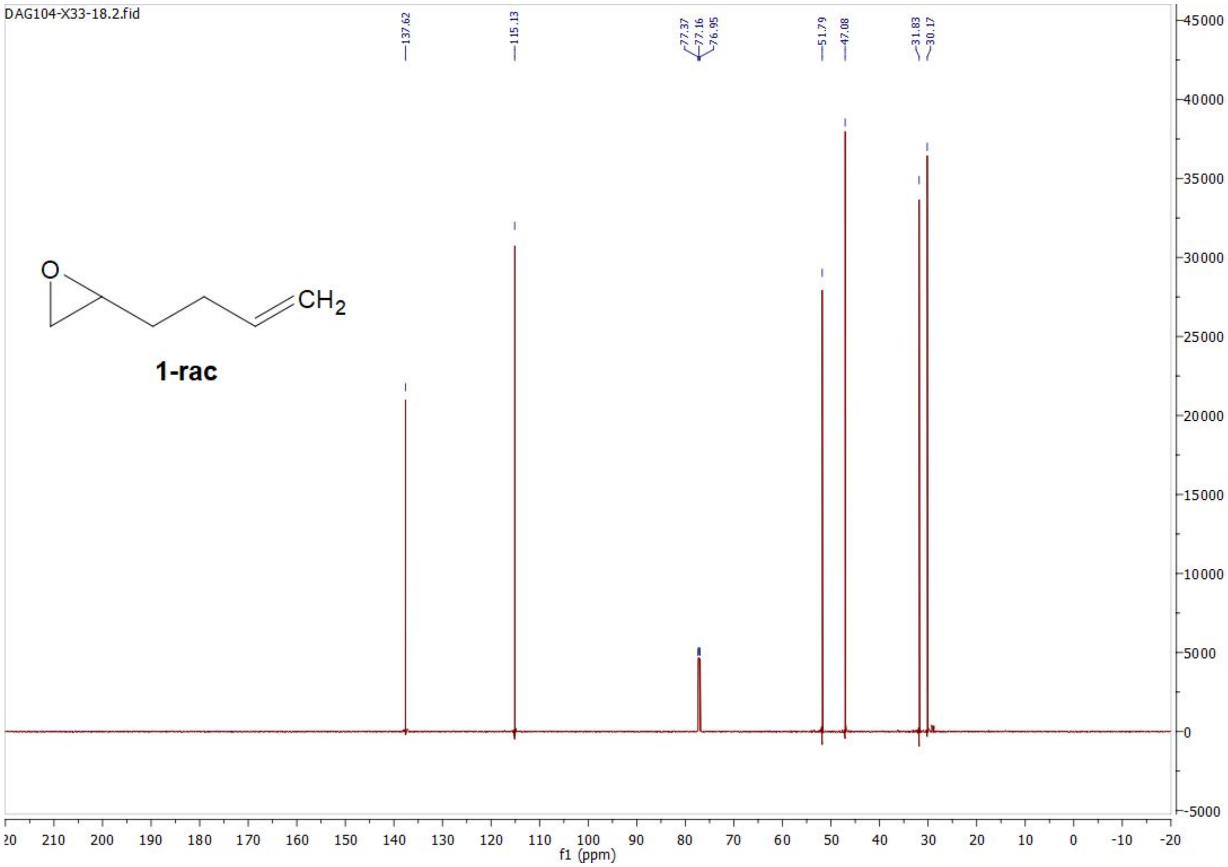

Figure S5. ^{13}CNMR (150MHz, $CDCl_3$) of **1-rac**

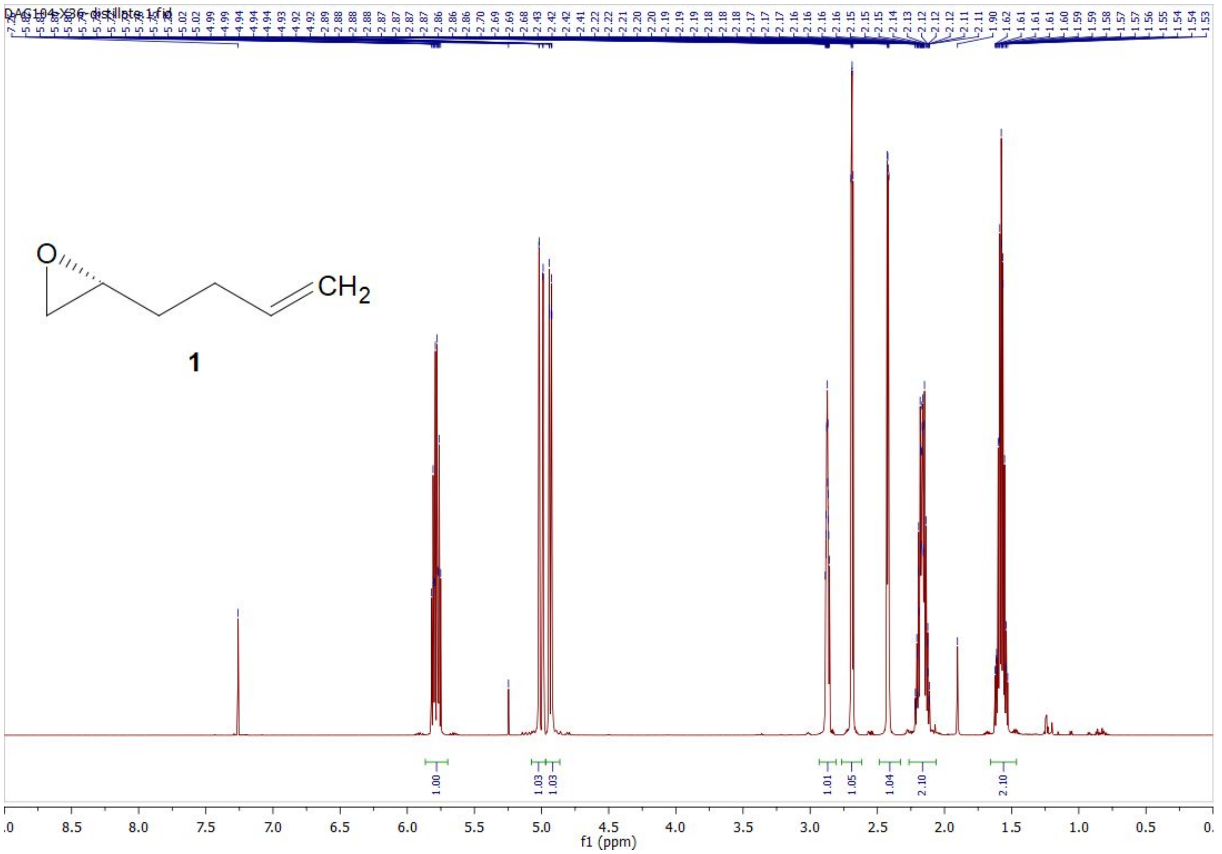

Figure S6. ^{1}HNMR (600MHz, $CDCl_3$) of **1**

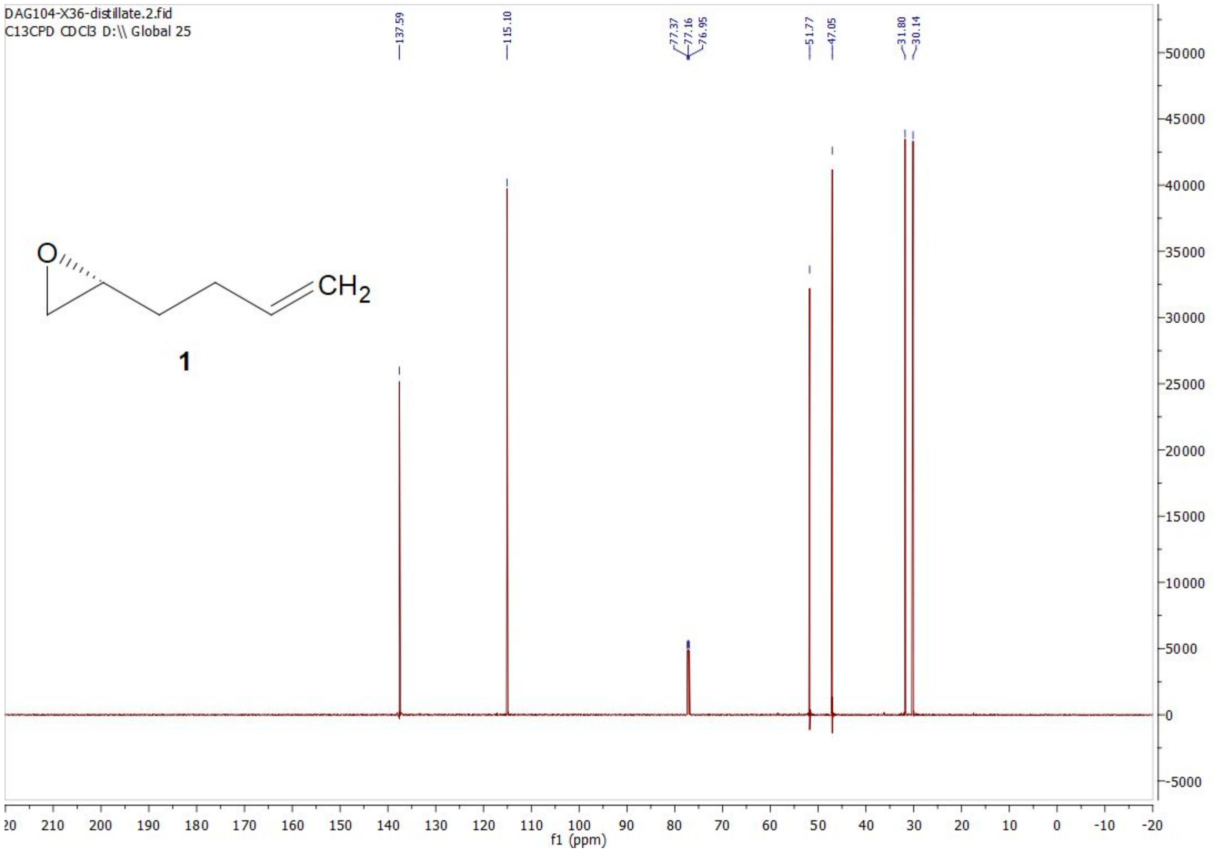

Figure S7. ^{13}CNMR (150MHz, $CDCl_3$) of **1**

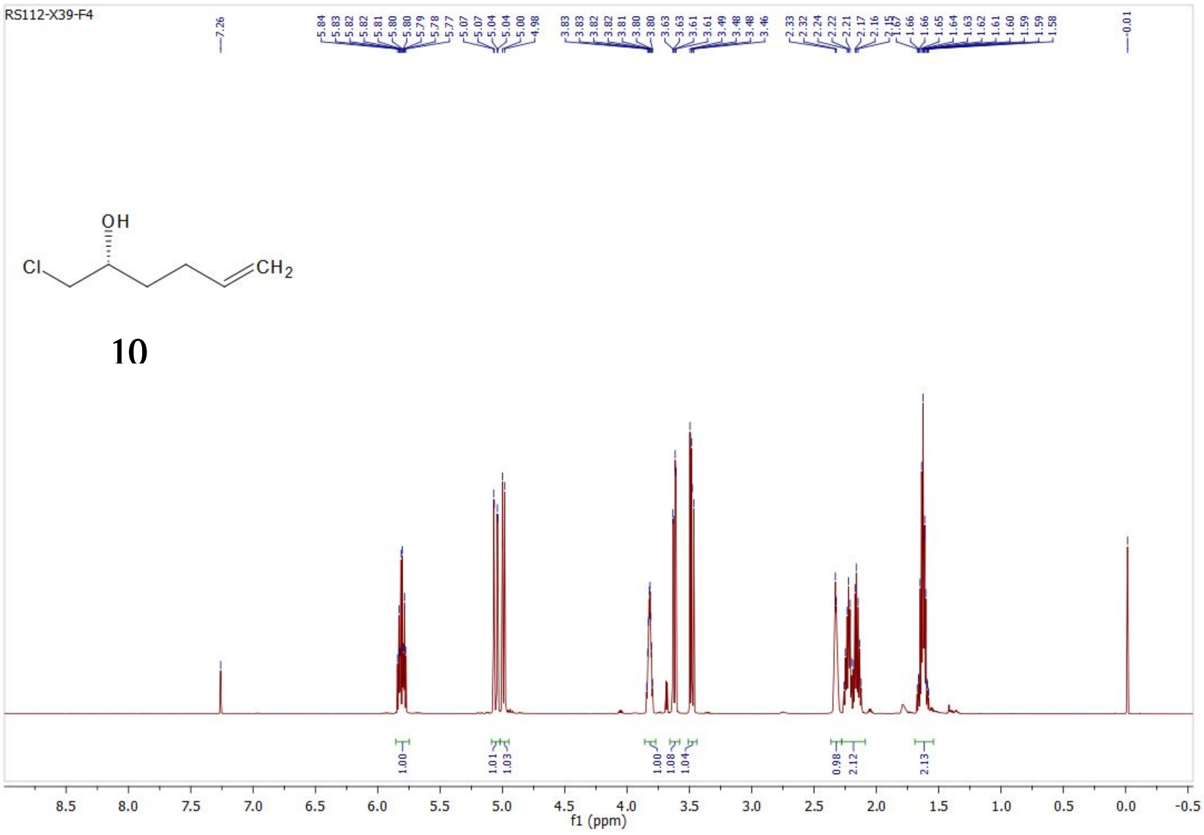

Figure S8. ^{1}HNMR (600MHz, $CDCl_3$) of **10**

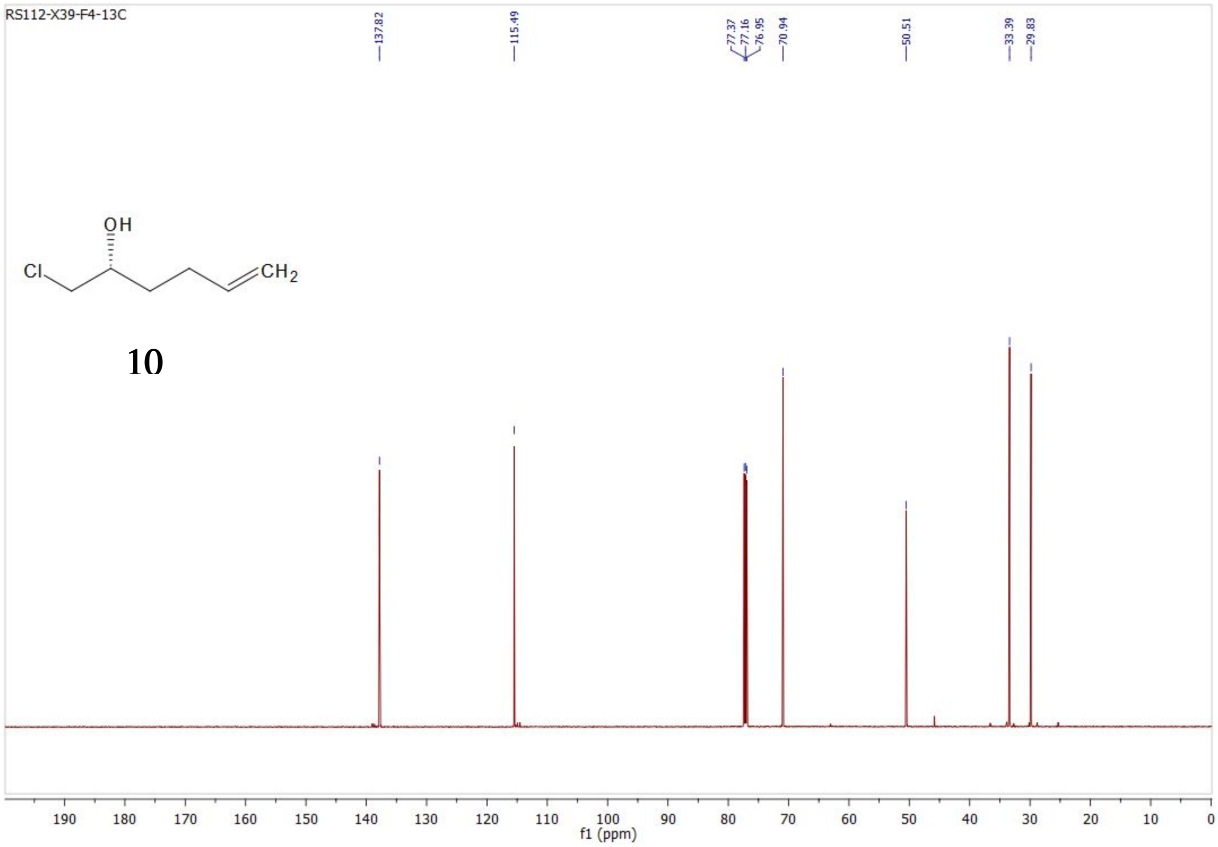

Figure S9. ^{13}CNMR (150MHz, $CDCl_3$) of **10**

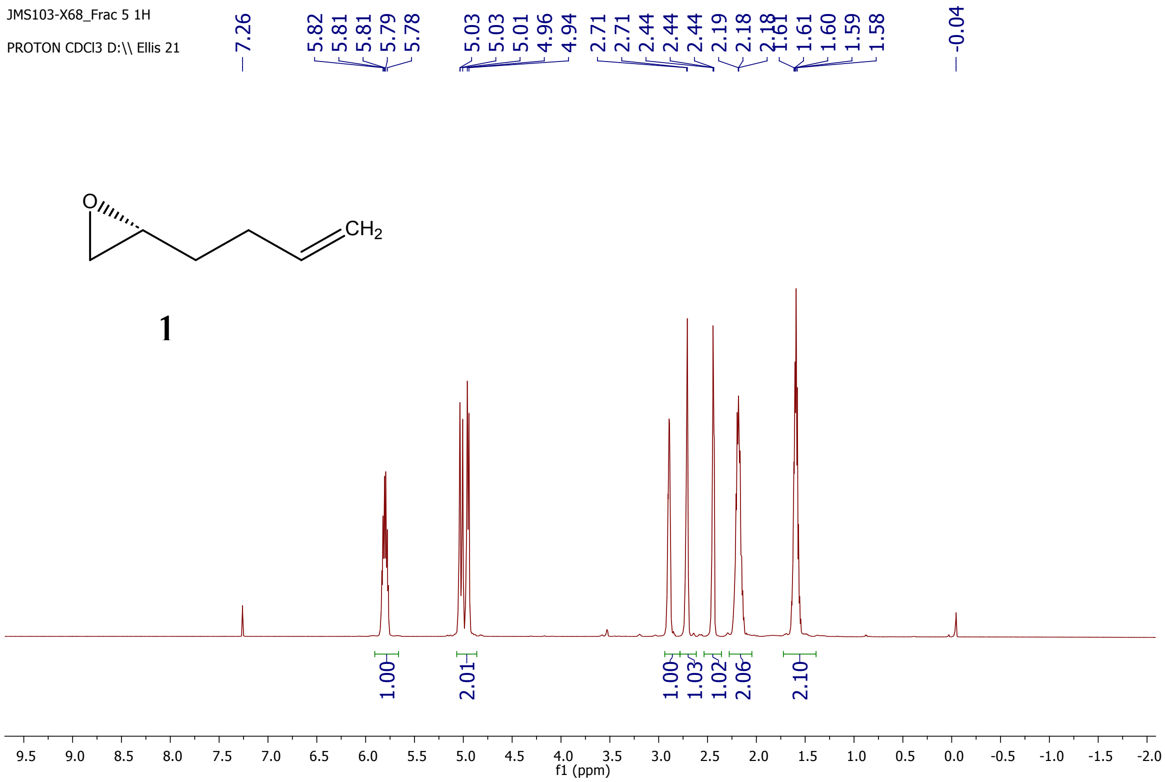

Figure S10. ^{1}HNMR (600MHz, $CDCl_3$) of **1**

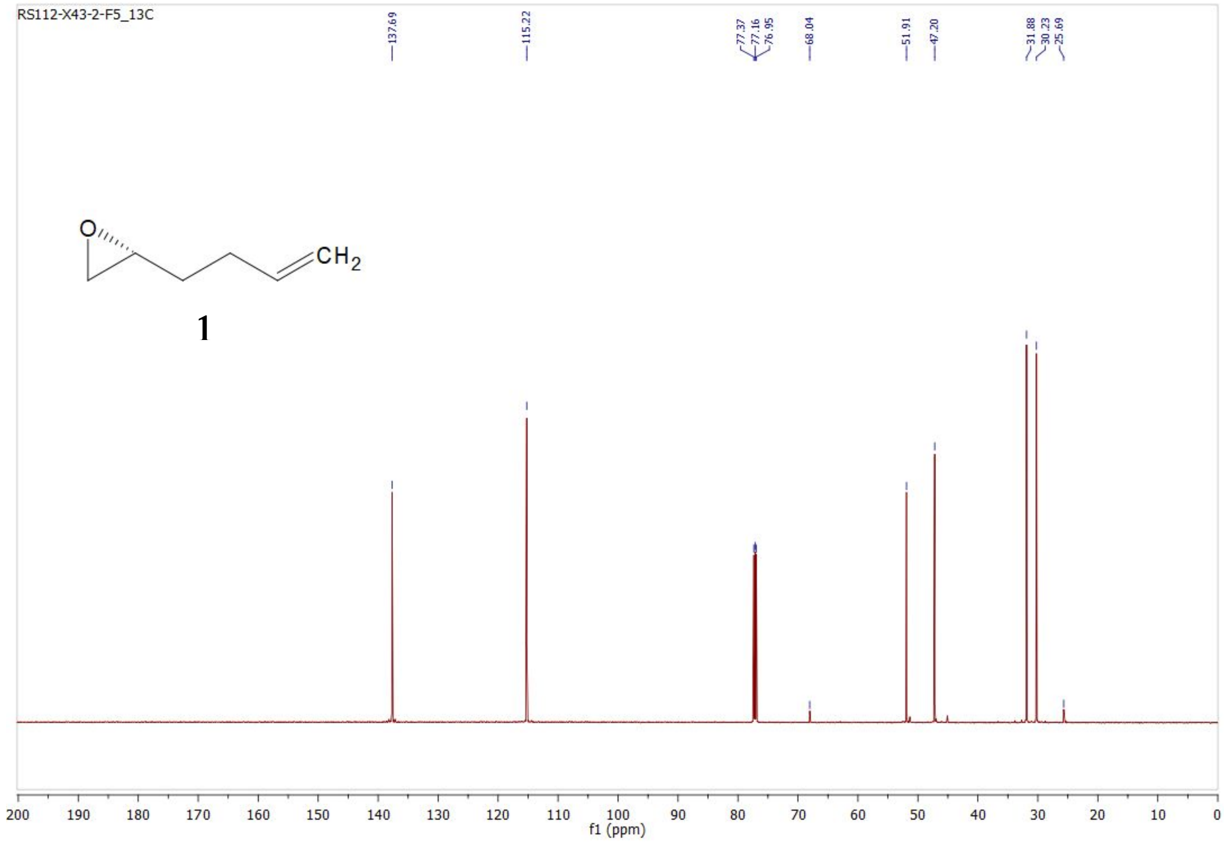

Figure S11. ^{1}HNMR (600MHz, $CDCl_3$) of **1**

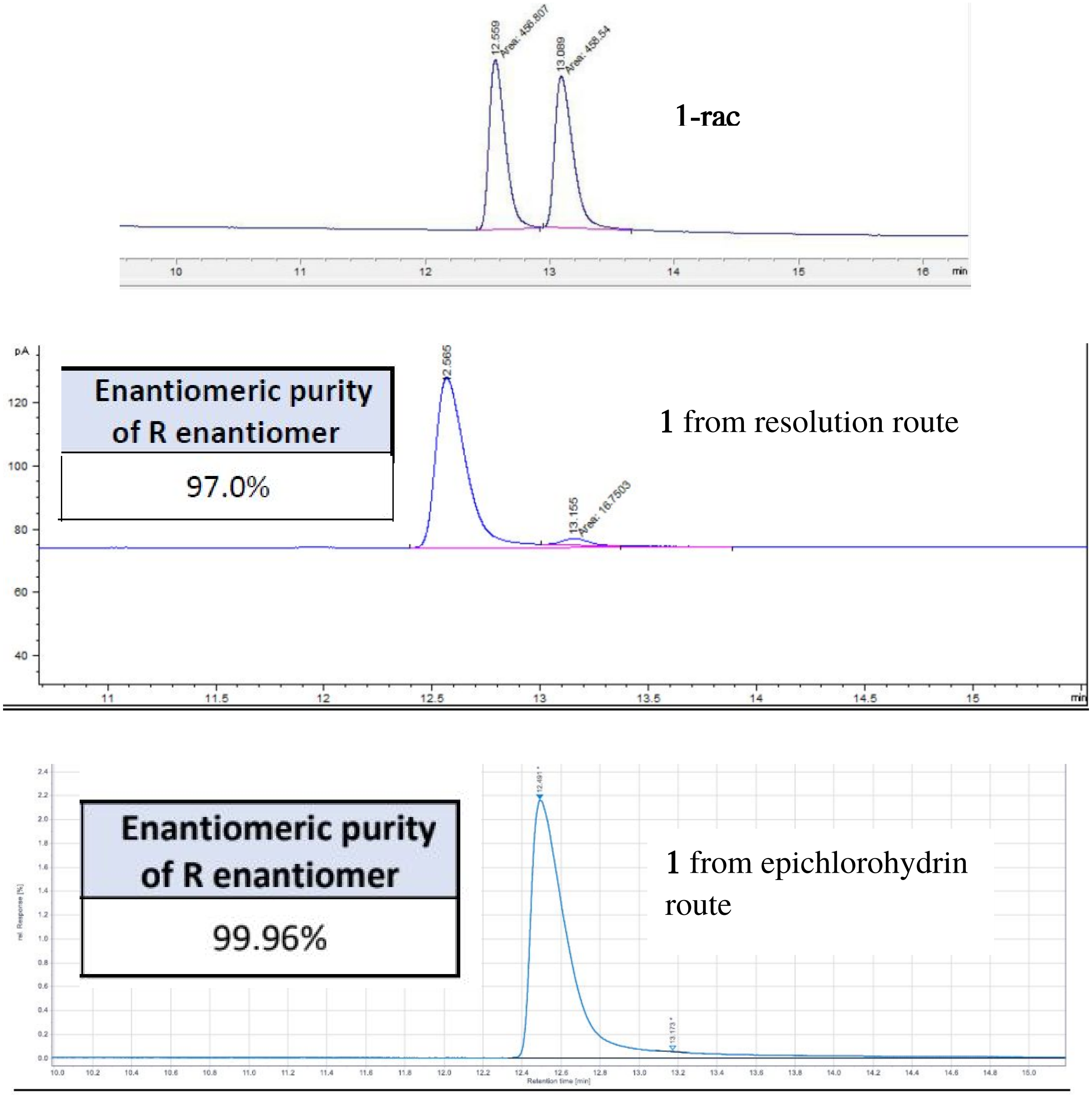

Figure S12. Chiral GC (GC-FID) spectrum of **1-rac, 1** (from resolution route), **1** (from epichlorohydrin route)
